# Hypoxia promotes a perinatal-like progenitor state in the adult murine epicardium

**DOI:** 10.1038/s41598-022-13107-2

**Published:** 2022-06-03

**Authors:** Angeliqua Sayed, Szimonetta Turoczi, Francisca Soares-da-Silva, Giovanna Marazzi, Jean-Sebastien Hulot, David Sassoon, Mariana Valente

**Affiliations:** 1 Université de Paris Cité, INSERM-U970, PARCC, 75006 Paris, France; 2 Lymphocytes and Immunity Unit, Immunology Department, Institut National de la Santé et de la Recherche Médicale U1223, Institut Pasteur, Paris, France; 3 CIC1418 and DMU CARTE, AP-HP, Hôpital Européen Georges-Pompidou, 75015 Paris, France

**Keywords:** Developmental biology, Multipotent stem cells, Regeneration, Stem-cell niche

## Abstract

The epicardium is a reservoir of progenitors that give rise to coronary vasculature and stroma during development and mediates cardiac vascular repair. However, its role as a source of progenitors in the adult mammalian heart remains unclear due to lack of clear lineage markers and single-cell culture systems to elucidate epicardial progeny cell fate. We found that in vivo exposure of mice to physiological hypoxia induced adult epicardial cells to re-enter the cell cycle and to express a subset of developmental genes. Multiplex single cell transcriptional profiling revealed a lineage relationship between epicardial cells and smooth muscle, stromal cells, as well as cells with an endothelial-like fate. We found that physiological hypoxia promoted a perinatal-like progenitor state in the adult murine epicardium. In vitro clonal analyses of purified epicardial cells showed that cell growth and subsequent differentiation is dependent upon hypoxia, and that resident epicardial cells retain progenitor identity in the adult mammalian heart with self-renewal and multilineage differentiation potential. These results point to a source of progenitor cells in the adult heart that can be stimulated in vivo and provide an in vitro model for further studies.

## Introduction

The epicardium gives rise to epicardial-derived cells (EPDCs) located in the subepicardial layer that migrate into the myocardium to give rise to interstitial cells, perivascular stroma and smooth muscle cells during development and early postnatal life^1–7^. The contribution of the epicardium to endothelial cells is controversial in mammals^1,5,8–12^ in part due to the small number of epicardial cells in the heart and a lack of suitable genetic cell fate models^8,13–15^. In addition, methodologies available to functionally study epicardial cells are limited to explant cultures that do not allow for definitive lineage and clonal analyses^16–21^.

In contrast to the adult, the neonatal heart is able to functionally recover following injury^22–24^. The perinatal heart is known to be hypoxic due to an immature vasculature structure^12,25–28^ and other studies have shown that many progenitors in different tissues are present in hypoxic niches^29^. These observations directly bear upon potential therapeutic avenues of discovery since it was reported recently that stepwise exposure of mice to hypoxia leads to a marked improvement in functional recovery of the heart following ischemia^30^. The degree of progenitor involvement in response to hypoxia in the adult remains unclear. The adult epicardium consists of progenitors and constitutes a physiological hypoxic niche due to the low capillary density and the constitutive expression of hypoxia inducible factor 1 alpha (HIF-1α)^31^.

While these findings show that hypoxia and signaling effectors in a hypoxic response play a role in epicardial development and cardiac injury^31–35^, it remains unknown whether there is a stimulation (priming) of the adult epicardium after in vivo hypoxia exposure. In addition, how prospectively isolated single epicardial cells behave is unknown, since efforts to date have been hampered by the inability to maintain and expand these cells in culture. Consequently, the functional analysis of epicardial cells, at the single cell level, in response to hypoxia has remained largely unaddressed. We therefore tested whether hypoxia regulates the epicardial progenitor competence. As a first step, we established a sorting strategy based upon known surface proteins together with a reporter mouse line for *Peg3/Pw1* (hereinafter referred to as *Pw1*). *Pw1* is expressed in a wide array of progenitor cells during development and in the adult, and is linked to the stem cell capacity of self-renewal as well as differentiation into specific tissue lineages^36–38^. We demonstrated previously that PW1 is expressed in a subset of stromal cells in the adult heart, which generates pro-fibrotic cells in response to injury^39,40^. However, PW1 expression had not been examined during early postnatal development when rapid heart growth and differentiation takes place. We show here that PW1 is expressed in the Gp38^+^ epicardium^41–43^ from development throughout adult life, consistent with progenitor capacity for this compartment^1–7^. We also show that hypoxia induces an increase in the number of PW1 expressing cells in the Gp38^+^ epicardium and PDGFRα^+^ subepicardium. Multiplex transcriptional profiling revealed that Gp38^+^PW1^+^ epicardial cells upregulate endothelial lineage-associated genes in response to hypoxia similar to the profile found in the epicardium at birth. Lastly, while previous studies relied on explants cultures, we show here that freshly purified Gp38^+^PW1^+^ epicardial cells display robust clonogenicity, self-renewal, and multipotency uniquely under hypoxic conditions, whereas they do not grow under normoxic conditions. Adult Gp38^+^PW1^+^ epicardial cells from physiological hypoxia-primed mice displayed a marked increase in their competence to grow and differentiate in vitro similar to what is observed with the neonatal epicardial cells. Taken together, our data support the hypothesis that the adult mammalian epicardium is a niche for resident Gp38^+^PW1^+^ progenitors and that exposure to hypoxia promotes robust progenitor activity similar to neonatal epicardium.

## Results

### Chronic physiological hypoxia exposure in the adult heart activates a developmental profile in the epicardial and subepicardial layers

**Figure 1:**
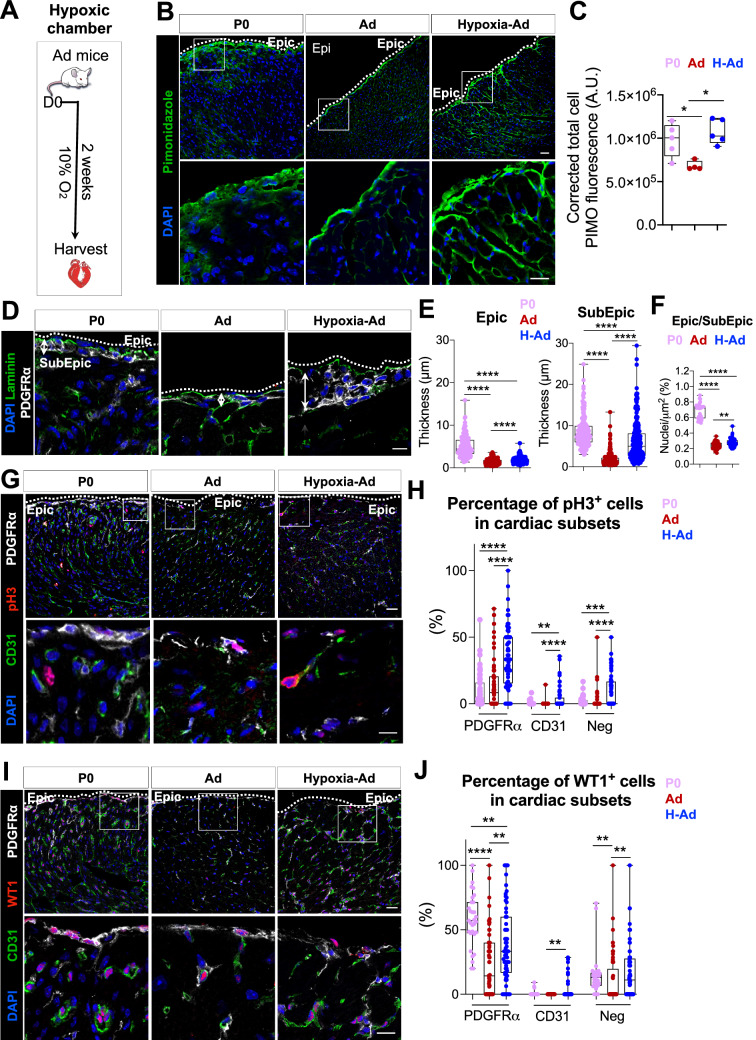
Two-weeks hypoxia exposure induces the reactivation of an epicardium-specific developmental growth program including proliferation and WT1 expression. (**A**) Schema of chronic hypoxia induction (10% O_2_) in the adult mice. (**B**) Hypoxic regions stain with Pimonidazole in P0, normoxic and hypoxic adult ventricles. Scale bar: 20 μm (top panels), scale bar: 10 μm (bottom panels). (**C**) Pimonidazole expression increases under hypoxia exposure (n = 3). (**D**) Epicardial and subepicardial layer determined by PDGFRα and Laminin staining in P0, normoxic and hypoxic adult ventricles. Scale bar: 10 μm. (**E**) Increase of epicardial and subepicardial thickness in hypoxic mice (n = 3, 20 images per heart, 5 measures per image and per layer). (**F**) Higher number of nuclei in hypoxic mice (n = 3, 20 images per heart). (**G**) Proliferative PDGFRα^+^ and CD31^+^ cells determined with pH3, in normoxic and hypoxic adult ventricles. Scale bar: 20 μm (top panels), 10 μm (bottom panels). (**H**) Percentage of proliferative cell increase in hypoxic mice (n = 3, 20 images per heart and per region). (**I**) Immunostaining of WT1, a marker of epicardial activation. Scale bar: 20 μm (top panels), 10 μm (bottom panel). (**J**) Percentage of WT1 expression increase particularly in the epicardial, subepicardial and endothelial cells in hypoxic mice (n = 3, 20 images per heart and per region). All nuclei were counterstained with DAPI. Dashed line delineates the epicardial (Epic) layer. Values are normalized by the total number of nuclei per layer and per field and all values are represented in percentage. The line in the box plot represents the median. Statistical significance was determined either by Mann–Whitney test or by one-way ANOVA (Kruskal–Wallis test) with uncorrected Dunn’s test, for 2 groups or 3 groups comparison, respectively. ****p < 0.0001, ***p < 0.001, **p < 0.01. *P0* postnatal day 0, *Ad* adult, *H* hypoxia, *Epic* epicardium, *SubEpic* subepicardium, *Neg* negative fraction.

Previous studies have shown that the epicardium is hypoxic^31^ and contains multipotent progenitors^43–46^. While there are conflicting reports regarding epicardial progenitor potential in the adult mammalian heart, it is well established that the epicardium has a pronounced progenitor capacity during mammalian development, corresponding to a hypoxic state in both the epicardium and underlying cell layers^47–50^. This raised the possibility that hypoxia directly regulates epicardial progenitors and is required for progenitor function. To explore the role of hypoxia, we placed adult mice into a hypoxic chamber at 10% O_2_ for 2 weeks (Fig. 1A), which is well tolerated and sufficient to induce overt physiological changes, including cardiac enlargement coupled with a reduced overall body weight, as reported by others^30^ (Supplementary Fig. IA,B in the Supplement). We used pimonidazole as a surrogate marker for hypoxic cells^51,52^. Pimonidazole staining in the epicardium and subepicardium (the outermost layers of the heart) as well as a stronger staining in the myocardial interstitium confirmed that the experimental conditions used were sufficient to induce generalized hypoxia in the heart (Fig. 1B,C) and we noted that pimonidazole labeling intensity was similar in to that detected at P0 (Fig. 1C).

Histological analyses revealed a striking increase in the thickness of the epicardial (PDGFRα^-^ outermost layer) and subepicardial (PDGFRα^+^ layer) layers in response to hypoxia (Fig. 1D,E) coupled with an increase in nuclei number (Fig. 1F), as observed during development (Supplementary Fig. IC,D in the Supplement). Consistent with these observations, we detected a marked increase in cell proliferation in subepicardial cells as both a percentage of the total population as well as in absolute number (PDGFRα^+^, Fig. 1G,H and Supplementary Fig. IIIE in the Supplement) in response to hypoxia using the cell proliferation marker phosphorylated histone H3 (pH3), suggesting that cell proliferation contributes to the increased thickness following hypoxia in the subepicardial layer (PDGFRα^+^ layer, Fig. 1D–F). While proliferative cells are present at low levels in the adult heart^53,54^ our results show that chronic exposure to hypoxia activates epicardial and subepicardial cell proliferation. Previous studies have shown that proliferation of epicardial/subepicardial cells is a feature of embryonic development^1–3,5,45^, therefore we tested whether other epicardial developmental markers were induced concomitant with cell proliferation. One such marker, WT1, is expressed in the epicardial and subepicardial compartments during embryonic heart development^5,13,55,56^. We observed a marked induction of WT1 expression in the epicardium and subepicardium (the most outer layers of the heart) as a percentage of the total population and absolute number (Fig. 1I,J and Supplementary Fig. IIIF in the Supplement). Taken together, our results show that physiological hypoxia activates both cell proliferation and WT1 expression in the adult that are hallmarks of early epicardial/subepicardial development.

### PW1 is expressed at high levels in the epicardium and subepicardium throughout life

**Figure 2:**
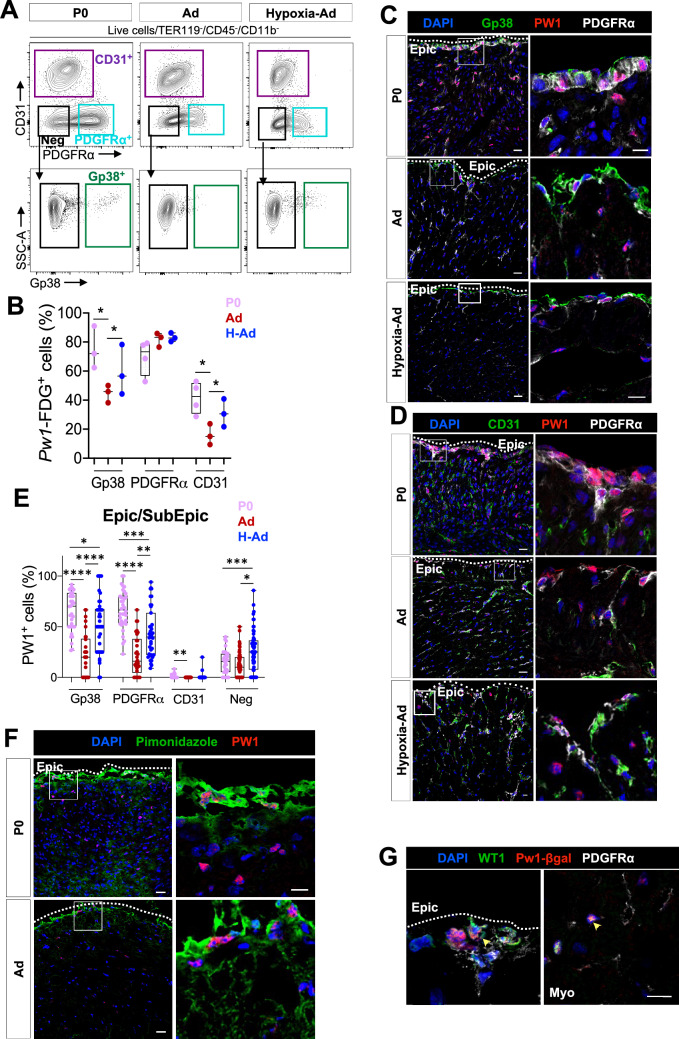
Adult mice exposed to chronic hypoxia (10%) upregulate PW1 expression in the activated epicardium and subepicardium. (**A**) Flow cytometry profiles and gating strategy of P0, Ad, and Ad-Hypoxia cardiac cell suspensions stained with CD31, PDGFRα and Gp38. (**B**) *Pw1*-FDG^+^ cells increase particularly in the endothelial and epicardial cell compartment after hypoxia induction in adult mice (n = 3). (**C**) Co-staining of PW1, Gp38 and PDGFRα in hypoxic mice (left panel). Scale bar: 20 μm (upper), 10 μm (bottom). (**D**) Co-staining of PW1, CD31 and PDGFRα in hypoxic adult ventricles. Scale bar: 10 μm. (**E**) Increase of PW1 expression in the epicardium (Gp38^+^) and subepicardium (PDGFRα^+^ and negative cell fraction) in hypoxic adult ventricle (n = 3, 20 images per heart and per region). (**F**) Co-expression of PW1 with pimonidazole in the epicardium and subepicardium in P0 and adult ventricles. Scale bar: 20 μm (left panels), 10 μm (right panels). (**G**) Co-staining of Pw1-β-gal with WT1 and PDGFRα in hypoxic adult mice. Scale bar: 10 μm. All nuclei were counterstained with DAPI. Values are normalized by the total number of nuclei per layer and per fields and all values are represented in percentage. Dashed line delineates the epicardial (Epic) layer. The line in the box plot represents the median. Statistical significance was determined by one-way ANOVA (Kruskal–Wallis test) with uncorrected Dunn’s test. ****p < 0.0001, ***p < 0.001, **p < 0.01, *p < 0.05. *Epic* Epicardium, *SubEpic* SubEpicardium, *Myo* myocardium, *P0* postnatal day 0, *Ad* adult, *H* hypoxia, *Pw1-FDG* *Pw1*-associated 5-Dodecanoylaminofluorescein Di-β-d-Galactopyranoside, *PIMO* Pimonidazole.

In order to isolate epicardial and subepicardial cells to assess their progenitor potential profile, we established a precise gating strategy to purify the different cardiac populations. We used antibodies to multiple cell surface proteins combined with a reporter mouse model for the expression of the adult progenitor cell marker *Peg3/Pw1* (hereinafter referred to as *Pw1*)^36–38^. Endothelial and stromal cells were defined by the expression of platelet endothelial cell adhesion molecule 1 (PECAM-1^+^ or CD31^+^, i.e. CD45^−^Ter119^−^CD11b^−^PDGFRα^−^CD31^+^) and platelet-derived growth factor receptor alpha (PDGFRα or CD140a, i.e. CD45^−^Ter119^−^CD11b^−^CD31^−^PDGFRα^+^), respectively. Hematopoietic cells were excluded by the combination of CD45, CD11b and Ter119 antibodies (Supplementary Fig. IIA in the Supplement). To define epicardial cells, we used an antibody to the surface glycoprotein 38 (Gp38 or podoplanin, Supplementary Fig. IIA in the Supplement), which has been shown to be expressed in the epicardial layer during development^41,42^ as well as in the adult^42,43^. While Gp38 is expressed in other cardiac cells population (hematopoietic, endothelial and stromal cells)^57–59^, we started by excluding hematopoietic cells (CD45^+^ CD11b^+^ Ter119^+^ fraction), endothelial cells (CD31^+^ fraction) and stromal cells (PDGFRα^+^ fraction) and defined our Gp38 epicardial population as CD45^−^CD11b^−^Ter119^−^CD31^−^PDGFRα^−^Gp38^+^ (hereinafter referred to as Gp38^+^ cells, Fig. 2A, Supplementary Fig. IIA,B in the Supplement). We found ~ 70% of the Gp38-expressing cells co-expressed the *Pw1* reporter gene at P0 and the *Pw1* reporter gene co-expression was maintained in the adult (≈ 50%, Fig. 2B). We observed that PDGFRα^+^ cells (subepicardial/stromal cells) expressed the *Pw1* reporter gene at all stages at high levels (≈ 80% and 60%, respectively, Fig. 2B), whereas CD31^+^ cells (endothelial cells) underwent a sharp decline in PW1 expression during postnatal life (P0 ≈ 45% vs. adult ≈ 10%, Fig. 2B). Flow cytometry analyses revealed that while the overall frequency of PW1^+^ cells increases in response to hypoxia (Supplementary Fig. IIIA,B in the Supplement), this increase is limited to the Gp38^+^ epicardial and CD31^+^ endothelial cells, whereas stromal cells do not show a significant change (Fig. 2B). Immunostaining confirmed PW1 protein expression in the epicardium (PDGFRα^−^ outermost layer) and subepicardium (PDGFRα^+^ layer) and showed that PW1^+^ cells co-expressed Gp38 in the epicardium and PDGFRα in the subepicardium (Fig. 2C,D). Although an overall ~ fourfold decrease of PW1 expression was observed between P0 and adult in the epicardium (Gp38^+^) and subepicardium (PDGFRα^+^), PW1 expression remained markedly higher in these layers (Fig. 2E). These results also reveal that subepicardial PDGFRα^+^ cells respond to hypoxia through an increase in PW1 expression, however the stromal PW1^+^PDGFRα^+^ compartment present in the cardiac interstitium do not show a significant response as measured by flow cytometry (Supplementary Fig. IIIC in the Supplement). We propose that the marked increase in PW1 expression in PDGFRα^+^ subepicardial cells is not observed using flow cytometry since the PDGFRα^+^ subepicardial cells constitute a minor population as compared to PDGFRα^+^ stromal cells which cannot be discriminated between the two compartments using the available cell surface markers.

We and others have shown that *Pw1* participates in multiple cell stress pathways^60–64^ and regulates glucose metabolism^65^. We therefore explored whether PW1 expression is regulated by hypoxia that is known to trigger cell stress and regulate glucose metabolism^66^. We confirmed that hypoxic cells are restricted to the epicardial and subepicardial layers in the adult (Fig. 1B) and express PW1 (Fig. 2F). Moreover, the developmental epicardial transcription factor WT1 was co-expressed with the *Pw1*-reporter gene in the epicardium (Gp38^+^), subepicardium (PDGFRα^+^, Fig. 2G and Supplementary Fig. IIID in the Supplement), indicating that the activated epicardial and subepicardial cells also co-expressed *Pw1*. Taken together, our data show that PW1 expression decreases during postnatal life in the ventricles, but levels remain elevated in the epicardial and subepicardial compartments. This observation is particularly relevant since epicardial and subepicardial layers have been shown to be a reservoir of cardiovascular progenitors during development^1–3,5,45^ and that PW1 is expressed by multiple progenitor cell types in the adult^36–38^.

### Epicardial cells express a progenitor/multipotent transcriptional profile in response to hypoxia

To better characterize the epicardial response to hypoxia and define how the adult epicardium compares to the perinatal state, we designed a multiplex qPCR panel of known genes corresponding to the major proposed epicardial lineages as well as to genes that respond to oxygen metabolism in freshly sorted cardiac populations (Supplementary Table I in the Supplement). Unsupervised hierarchical clustering (Supplementary Fig. IVA in the Supplement) and UMAP1 vs. UMAP2 (Uniform Manifold Approximation and Projection, Supplementary Fig. IVB in the Supplement) at population level segregated the main cardiac populations in: CD31^+^ endothelial cells irrespective of the time-point or hypoxia exposure (cluster I); developing Gp38^+^ epicardial cells (E17.5 and P0, cluster II); the majority of PDGFRα^+^ cells assembled in two adjacent clusters (clusters III and IV), which differ from each other due to high expression of *Cdh5* and lower levels of *Col1a1*, *Tcf21* and *Postn* in the cluster of hypoxia-primed adult PDGFRα^+^ and Gp38^+^ epicardial cells (cluster IV, Supplementary Fig. IVA,B in the Supplement); cluster V is composed of adult Gp38^+^ epicardial cells (both normoxic and hypoxic) together with hypoxia-primed PDGFRα^+^ and CD31^+^ cells (Supplementary Fig. IVA,B in the Supplement). We also tested the expression of two mesothelial genes *Upk1b* and *Upk3b*, and observed that their expression was restricted to Gp38^+^ cells (Supplementary Fig. IVC in the supplement). While these results validate our approach in discriminating the major cardiac populations, they reveal a clustering of Gp38^+^ cells with other cell types, reflecting that Gp38^+^ cells transcriptional profile overlap of hypoxia exposed epicardial, stromal, and endothelial cells (Supplementary Fig. IV in the Supplement).

**Figure 3:**
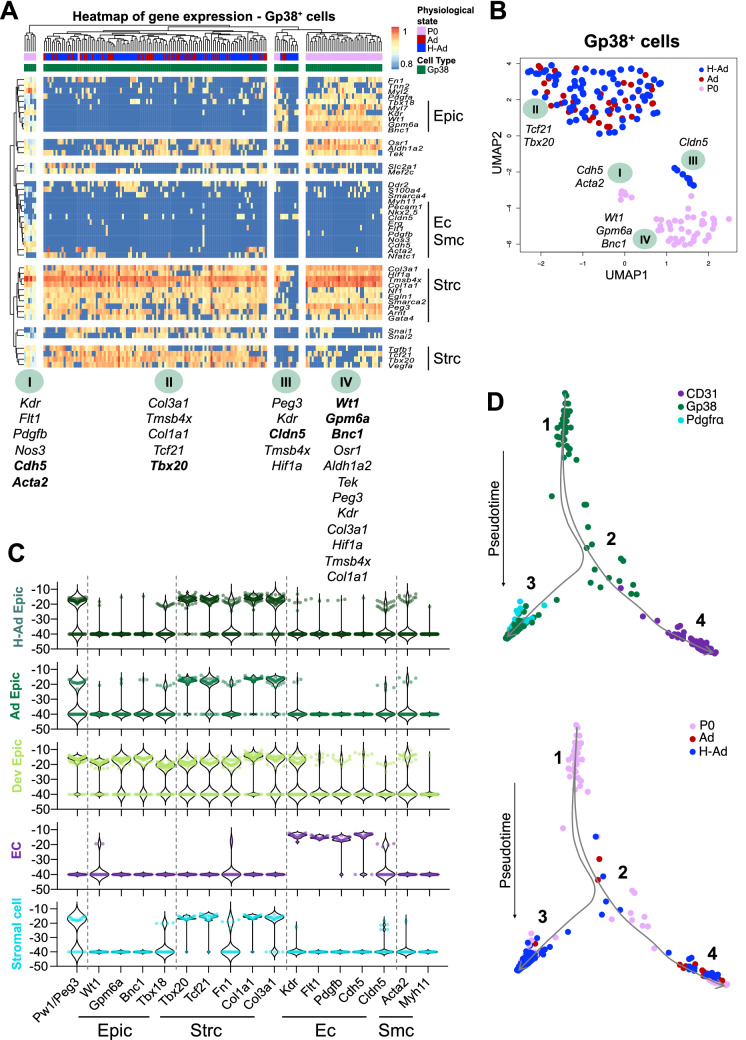
Transcriptional profiling demonstrates the activation of the epicardial developmental growth program in response to chronic hypoxia and a lineage overlap with PDGFRα^+^ and CD31^+^ cells. (**A**) Multiplex qPCR heat-map at single cell level reveals different developmental states of the Gp38^+^ epicardial compartment from P0, Ad (adult) and H-Ad (hypoxic adult). Each column represents one cell with the corresponding color-code according cell type and physiological state. Hierarchical clustering reveals 4 major group of cells labeled I, II, III, IV with the differentially expressed genes listed below. A total of 168 cells were analyzed. Gene expression was normalized to *Gapdh*, and unsupervised hierarchical clustering was performed. (**B**) tSNE analysis of multiplex qPCR from 168 epicardial cells (P0, Ad and H-Ad). Genes displayed are the most differential expressed genes corresponding to each cluster and are color-coded according to the to the physiological state. (**C**) Violin plot of genes corresponding to the major epicardial derived lineages (Epic: epicardium, Sc: Stromal cells, Ec: endothelial cells, Smc: smooth muscle cells) of *Pw1/Peg3* and of metabolic pathways from a total of 186 cells analyzed. (**D**) Diffusion map (pseudo-time analysis) of Sc, Ec, and Smc populations based on single cell gene expression. Color-code according to the cell type (top panel) and according to the physiological state (bottom panel). *P0* postnatal day 0, *Ad* adult, *H* hypoxia, *Epic* epicardium, *Ec* endothelial cell, *Smc* smooth muscle cell, *Strc* stromal cells.

We analyzed 382 cells at the single cell level, and further confirmed our results observed at the population level (Supplementary Fig. IV in the Supplement) by unsupervised hierarchical clustering (heat-map, Supplementary Fig. VA in the Supplement) and tSNE projection (t-Distributed Stochastic Neighbor Embedding, Supplementary Fig. VB in the Supplement). Freshly sorted Gp38^+^ cell compartment is composed of epicardial cells in different developmental states and were split in the four obtained clusters (Supplementary Fig. V in the Supplement). Further single cell transcriptional analysis of the Gp38^+^ epicardial population (168 cells) revealed unique transcriptional signatures of the different epicardial subsets (Fig. 3A). Epicardial-associated genes (*Wt1*, *Gpm6a*, *Bnc1* and *Tbx18*) are highly expressed during development (P0 cluster I, III and IV) and after injury^5,13,55,56^. We observe a re-activation of these genes after hypoxia exposure in a fraction of cells at the transcriptional level (cluster III, Fig. 3A), as well as at the protein level (Fig. 1I,J). Cluster I encompass a compartment of P0 epicardial cells expressing the epicardial specific genes together with *Kdr*, *Flt1*, *Pdgfb*, *Nos3*, *Cdh5* and *Acta2*. Co-expression in the same cell of epicardial, endothelial and *Acta2* is compatible with an epicardial commitment to the endothelial-like lineage. Cluster II contains the majority of adult normoxic and hypoxic Gp38^+^ epicardial cells expressing stromal associated genes (*Col3a1*, *Tmsb4x*, *Col1a1*, *Tcf21* and *Tbx20*), confirming their lineage relationship as shown by others^1–3,5,45^. Cluster III and IV show high levels of epicardial specific genes (*Wt1*, *Gpm6a*, *Bnc1* and *Tbx18*) indicative of the most immature epicardial profile. Cluster III differs from cluster IV due to the presence of hypoxia-primed epicardial cells and the additional expression of *Cldn5*. This cluster also diverges from cluster II, where the majority of the hypoxia-primed epicardial cells are, due to the reduction of the fibrotic genes (*Tcf21, Tbx20, Tgfb1* and *Col1a1*, Fig. 3A). The projection of the transcriptional data as UMAP1 vs. UMAP2 (Uniform Manifold Approximation and Projection, Fig. 3B) show similarly to the heatmap clustering (Fig. 3A), with 4 clusters of epicardial cells. The UMAP analysis highlights the similarities between cluster I (P0 epicardial cells) and cluster III (P0 and hypoxia-primed epicardial cells) with the differential expression of genes associated to the endothelial lineage (Fig. 3B).

Based on single cell data, gene expression profiles corresponding to the major epicardial derived lineages, i.e. epicardial, stromal and smooth muscle cells were analyzed, together with the endothelial lineage genes (Fig. 3C). While the developing epicardium shows a mixed profile with cells expressing genes characteristic of all lineages (epicardial, stromal, endothelial and smooth muscle cells), the adult epicardial layer only expresses epicardium and stromal cell transcripts. In contrast, hypoxia-primed adult epicardium up-regulates the expression of smooth muscle-associated genes in a subset of cells comparable to the developing epicardium profile (Fig. 3C). We observed that epicardial genes were up-regulated in a small fraction of cells, and that *Tbx18* showed a more marked increase in expression after hypoxia stimulus (Supplementary Fig. VIA in Supplement). Although few cells up-regulate the expression of endothelial genes (Fig. 3C), we observed a significant increase in the relative expression of several genes underlying angiogenesis (*Kdr*, *Flt1*, *Pecam-1* and *Pdgfb*) in this small subset (Supplementary Fig. VIB in Supplement).

A determination of lineage trajectories, while not definitive, can be proposed based upon multiplex single-cell experimental tools and diffusion maps (or pseudotemporal ordering) as shown by others^67^. We therefore generated diffusion maps for the 3 populations examined here. Similar to our observations with unsupervised clustering and tSNE analyses (Fig. 3A,B), we found that Gp38^+^ epicardial cells were more dispersed, whereas PDGFRα^+^ stromal and CD31^+^ endothelial cells were found closer together (Fig. 3D). The resulting trajectory initiates with the most immature phenotype (Gp38^+^ cells, root, labeled 1) and splits into two distinct cell fates, the stromal cells (labeled 3) and endothelial cells (labeled 4). Perinatal epicardial cells (P0) are positioned at the root of the diffusion map (Fig. 3D, labeled 1), followed by a split node (Fig. 3D, labeled 2) into two branches: one of PDGFRα^+^ cells (Fig. 3D, labeled 3); and another of CD31^+^ endothelial cells (Fig. 3D, labeled 4). We noted a large overlap between the PDGFRα^+^ cell branch and the Gp38^+^ epicardial cells consistent with a cell fate lineage relationship. Endothelial cell branch shows less overlap and is more clearly segregated from Gp38^+^ cells. However, we noted a fraction of hypoxia-primed adult Gp38^+^ epicardial cells (ratio of 1/12 cells) that cluster together with a fraction of P0 epicardial cells (ratio of 1/5 cells, Fig. 3D, labeled 2) alongside the pathway of the endothelial cells fate. This result indicates that epicardial cells are distinct from the endothelial compartment. Overall, Gp38^+^ cell compartment is composed of epicardial cells in different developmental stages, showing different transcriptional profile for the genes analyzed consistent with the proposal that this compartment consists of epicardial cells and epicardial-derived cells. As expected, P0 derived epicardial cells have the most immature transcriptomes. A significant fraction of epicardial cells, irrespective of the stage or conditions from which they were isolated largely overlap with the PDGFRα^+^ population which is in agreement with previous demonstrations of a lineage relationship between the epicardium and stromal/fibroblast cells^1–7,46,68–72^. We observed that a distinct subset of P0 (1/5 cells) and hypoxia-primed (1/12 cells) epicardial cells cluster together with the endothelial population, highlighting similarities between the transcriptional profile of these cells. Additionally, hypoxia-primed epicardial cells showed a 1.6-fold increase in the frequency of cells that clustered with endothelial cells compared to adult normoxic cells (1/19). We note that we did not observe a complete endothelial transcriptional program in these cells, however the data suggest that the adult epicardium is activated to give rise to a population of cells with an endothelial-like cell fate potential in response to hypoxia, which is similar to what is observed during perinatal development^7,73^.

### The epicardial in vitro stem cell potential is dependent upon hypoxia

**Figure 4:**
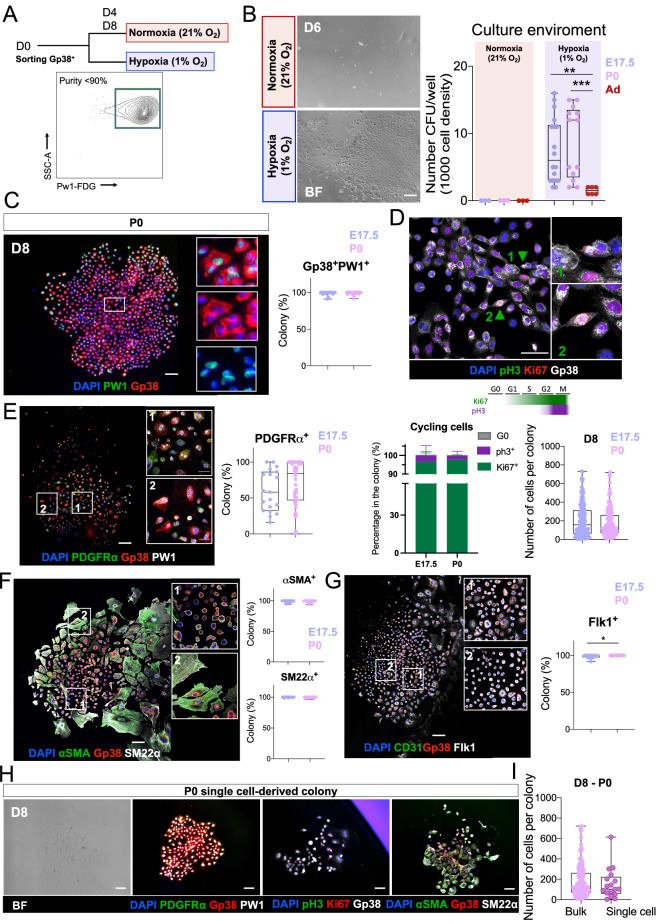
In vitro hypoxia maintains growth and cell fate potentials to Gp38^+^PW1^+^ epicardial cells. (**A**) Schema of the in vitro experiment: cell sorting (Gp38^+^*Pw1*-FDG^+^ cells) with a purity of more than 90% from E17.5, P0, adult (Ad) and in vivo hypoxia-primed adult (H-Ad) hearts. Freshly sorted cells were cultured from D0 to D8 either in normoxia (21% O_2_) or hypoxia (1% O_2_). (**B**) Frequency of colonies (1000 cells/well) cultured either under normoxia or hypoxia in vitro condition and the respective brightfield pictures of the cobblestone-like Gp38^+^ colonies. (**C**) Immunolabeling shows the colonies keep the Gp38 and Pw1 expression similar to their initial ex vivo profile (clonogenicity). (**D**) Immunolabeling of the cell cycle makers Ki67 and pH3 highlights the proliferative profile (green arrowheads) of colony of Gp38^+^PW1^+^ cells (self-renewal). (**E**) Epicardial-derived and stromal cell markers (Gp38 and PDGFRα) are observed in the main cell fraction of the colony. (**F**) Smooth muscle cell markers (αSMA and SM22α) are observed in the majority of the cells composing the Gp38^+^ colony. (**G**) Immature endothelial marker Flk1, but not CD31, is observed to be expressed in vitro in Gp38^+^ colonies. (**H**) Single cell-derived colonies confirm the multipotential ability of the Gp38^+^PW1^+^ cells to express proteins of smooth muscle, stromal and immature endothelial cell. Scale bar: 100 μm. All nuclei were counterstained with DAPI. Statistical significance was determined by Mann–Whitney test. ***p < 0.001, **p < 0.01, **p < 0.05. *BF* Brightfield, *E.17.5* embryonic day 17.5, *P0* postnatal day 0, *Ad* adult.

The cell fate potential of the epicardium can only be defined using reliable lineage markers which are presently unavailable. This limitation is further compounded by the currently available in vitro assays to study epicardial cells biology that are based on tissue explants and migratory potential of epicardial-derived cells^16–21^. Tissue explant assays do not allow for the determination of the main stem cell properties (i.e. self-renewal, clonogenicity and multipotency) and bias the study to cells able to migrate out of the explant. Our data reveals that epicardial cells are activated in response to hypoxia, raising the possibility that these cells require a hypoxic environment. Using our sorting strategy (Supplementary Fig. IIA in the Supplement), we prospectively isolated and freshly cultured Gp38^+^PW1^+^ epicardial cells from fetal (E17.5), newborn (P0), and adult hearts both in normoxic (21% O_2_) and in hypoxic (1% O_2_) conditions (Fig. 4A). We found that Gp38^+^PW1^+^ epicardial cell growth was completely dependent upon hypoxic conditions regardless of their developmental state, whereas these cells did not expand in normoxic conditions and only few cells were present following several days in culture (Fig. 4A,B). We noted that adult-derived epicardial cells displayed a significant decrease in the ability to grow in culture (Fig. 4B). Because the ability of adult epicardial cells to grow in culture is decreased and the frequency of epicardial cells recovered from an adult heart is substantial smaller, we characterized the in vitro epicardial colonies potential using the developing epicardial Gp38^+^ cells (E17.5 and P0).

Low-density plating assays showed that Gp38^+^PW1^+^ epicardial cells form colonies with an epithelial-like honeycomb morphology (Fig. 4B,C). The developing (E17.5/P0) colonies displayed complete overlap of Gp38 and PW1 expression (Fig. 4C), showing that hypoxia maintains their original profile (Fig. 4A). Cell proliferation was assessed by Ki67 and pH3 expression and showed that ~ 95% cells (Gp38^+^/Ki67^+^) were proliferative of which 5% were actively dividing (Gp38^+^/pH3^+^, Fig. 4D and Supplementary Fig. VIIA in the Supplement). This profile was observed at D4 or D8 in culture (Supplementary Fig. VII in the Supplement), confirming that colony growth is due to proliferation of Gp38^+^ cells. Additionally, colonies shared a similar average size when derived from E17.5 and P0 (Fig. 4D, right graph) revealing a similar growth potential in cells derived from early stages. We next characterized the colonies for each relevant epicardial lineage following 8 days in culture. We observed that PDGFRα was expressed in a subset of the colony (Fig. 4E and Supplementary Fig. VIII in the Supplement) and its expression is acquired by D4 (Supplementary Fig. VII in the Supplement). This result is consistent with previous observations that PDGFRα is expressed in epicardial-derived cells found in the subepicardial zone (Fig. 2C,D). The Gp38^+^PW1^+^ colonies also showed expression of the smooth the muscle cell markers αSMA and SM22α (Fig. 4F and Supplementary Fig. VIII in the Supplement). The majority of cells within the colony expressed both proteins, however the co-expression of αSMA was associated with a smooth muscle-like cell morphology. We detected that colonies typically consisted of a centrally located core of cells that were round, compact and expressed high levels of Gp38^+^ (Fig. 4F, inset 1). This core was typically surrounded by an outer layer of differentiating αSMA^+^ cells with a lower nucleus/cytoplasm ratio and lower levels of Gp38 expression (Fig. 4F, inset 2). Furthermore, we observed a widespread expression of Flk1, an early endothelial commitment marker (Fig. 4G and Supplementary Fig. VIII in the Supplement). While Flk1 expression was observed in Gp38^+^ cells, we did not observe CD31 expression, indicating that conditions used in our culture conditions supports cell expansion, but are not optimized for full endothelial differentiation consistent with previous studies that have shown that endothelial differentiation requires specific cytokines as well as high density and specific substrate conditions^74,75^, which we could not used in tandem with our colony formation assays. We further confirmed the ability to form colonies at the single cell level (Fig. 4H,I), and the capacity of Gp38^+^Pw1^+^ cells to express proteins associated with smooth muscle, stromal, and immature endothelial cells (Fig. 4H). Taken together, we describe novel culture conditions that allow for the maintenance and expansion of Gp38^+^PW1^+^ epicardial cells using hypoxic conditions and that these cells are multipotent progenitors that express key markers that are associated with stromal, smooth muscle, and immature endothelial cell fates.

### Physiological hypoxic priming induces the capacity to form CFU in adult epicardial cells

**Figure 5:**
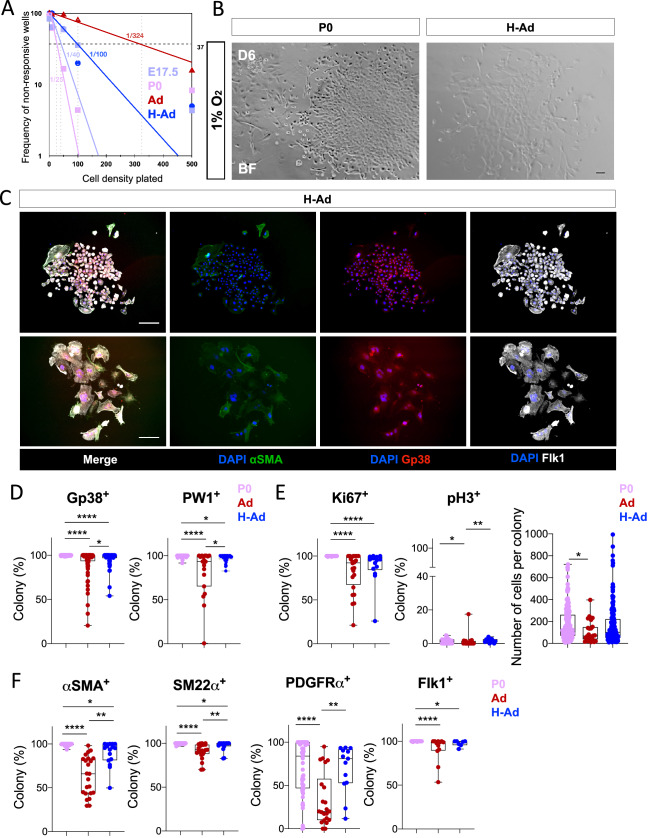
In vivo hypoxia-priming confers/restores the CFU ability in adult epicardial cells. (**A**) Limiting dilution assay to determine (at the maximum likelihood parameter) the frequency of stem cells (CFU) within the Gp38^+^PW1^+^ cell population. The graph shows the number of negative wells (non-response) for the growth of a colony and the CFU probability (E17.5—1/40, P0—1/25, Ad—1/324 and H-Ad—1/100). (**B**) Gp38^+^ cells form under hypoxia a cobblestone structure typical of epithelial structure colony (H-Ad), similarly to that observed in developing epicardium (E17.5 and P0). (**C**) Immunolabelling of the hypoxia-primed adult Gp38^+^ colonies for relevant proteins of clonogenicity/self-renewal and multipotency. (**D**) Comparison of the Gp38 and PW1 protein expression (clonogenicity/self-renewal) in P0, adult (normoxia and hypoxia). (**E**) Comparison of the Ki67 and pH3 cell cycle protein (self-renewal) in P0, adult (normoxia and hypoxia). (**F**) Comparison of the αSMA, SM22α, PDGFRα and Flk1 protein expression (multipotency) in P0, adult (normoxia and hypoxia). Scale bar: 100 μm. All nuclei were counterstained with DAPI**.** The Maximum likelihood estimation (MLE) test (ELDA assay) was used for the statistical determination of the CFU values for each stage of Gp38^+^ cells with a *p* < 0.0001. The line in the box plot represents the median. Statistical significance was determined by one-way ANOVA (Kruskal–Wallis test) with uncorrected Dunn’s test. ****p < 0.0001, **p < 0.01, *p < 0.05. *P0* postnatal day 0, *Ad* adult, *H* hypoxia.

Gp38^+^PW1^+^ epicardial cells constitute a minor overall cell population in the adult heart (Fig. 2A) and the epicardium is relatively quiescent^5,13,55,56^ but hypoxic^31^. This raised the question whether in vivo hypoxia priming directly regulates epicardial progenitors. We compared the potential of Gp38^+^PW1^+^ epicardial cells derived from E17.5, P0, adult normoxia or adult hypoxia-primed mice. Limiting dilution analysis showed that P0 and E17.5 gave rise to epicardial colonies at similar frequency (1/25 and 1/40, respectively). Nevertheless, P0-derived Gp38^+^PW1^+^ epicardial cells showed a higher capacity to form colonies (CFUs) as compared to cells derived from E17.5 (Fig. 5A), which may reflect that the epicardium is more active during early postnatal growth when the bulk of coronary vasculature is formed/matured^76^. Moreover, adult epicardial cells from normoxic mice showed an eightfold and 13-fold lower CFU capacity (1/324) as compared to fetal and newborn derived epicardial cells, respectively (Fig. 5A). We therefore tested the capacity of Gp38^+^PW1^+^ epicardial cells derived from adult hearts following exposure to physiological hypoxia to test whether the level of oxygen accounted for CFU capacity. We found that the hypoxic ‘priming’ in vivo prior to culturing freshly isolated epicardial cells induced a threefold increase in the colony formation in vitro (Fig. 5A,B). Furthermore, we found that the colonies derived from adult hypoxia-primed epicardial cells displayed a higher frequency of dividing cells (pH3^+^) as well as of PDGFRα^+^ cells similar to the behavior of colonies from newborn (P0) mice (Fig. 5C–F), when the Gp38^+^PW1^+^ epicardial cells were more active. In conclusion, these observations demonstrate that hypoxia provides a critical stimulus for epicardial cell growth, expansion and differentiation and that the low oxygen levels exposure in vivo play a critical role in promoting these activities.

## Discussion

The epicardium is a mesothelial layer that surrounds the heart of all vertebrates and gives rise to multiple cell lineages during heart development and regeneration. There is considerable research effort aimed at identifying factors that activate the adult epicardium in order to promote a beneficial recovery following cardiac injury^43,68,77–83^. The embryonic and fetal hearts are physiologically hypoxic due to an underdeveloped cardiac vascular system and placenta restriction^12,25–28,49,50,84,85^, whereas the hypoxic state is only maintained in the adult mammalian epicardium, as shown in this study and by others^31^. The epicardium has been shown to drive heart morphogenesis and serve as a reservoir of progenitors of non-cardiomyocyte lineages throughout life and in response to injury in non-mammalian vertebrates^1–7^. The progenitor capacity of the adult mammalian epicardial cells is less explored, but is considered to be less robust. Additionally, the contribution of the epicardium to coronary endothelial cells in mammalian hearts is controversial and it remains unclear if this property is maintained in the adult.

**Figure 6:**
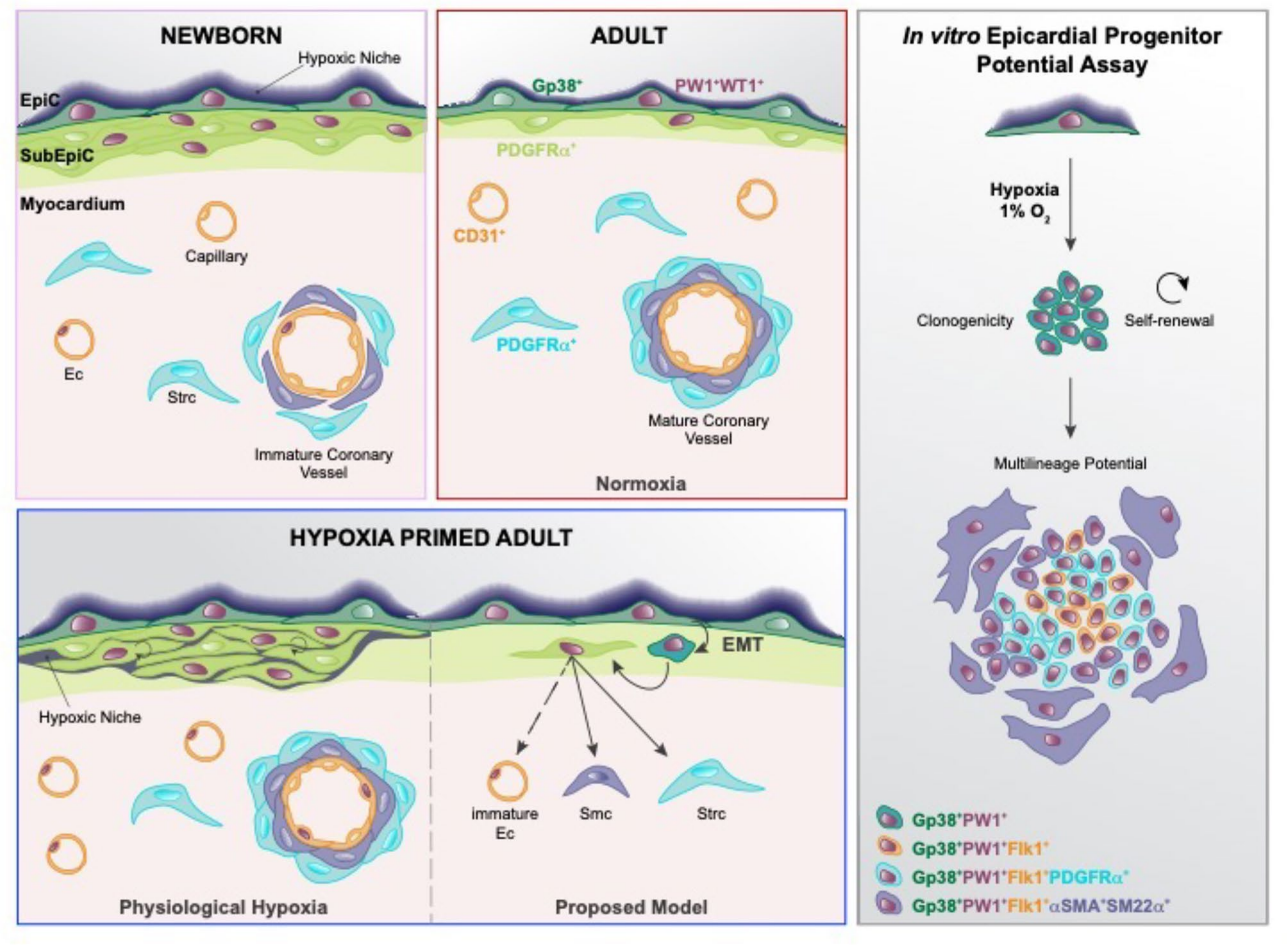
Proposed model for hypoxia activation of the adult murine epicardium to a progenitor cell function and multipotency. Epicardium is an active reservoir of progenitor cells that gives rise to coronary vasculature and stroma cells during perinatal life. The adult mammalian epicardium is a heart resident progenitor niche that maintains PW1 and WT1 expression, and a hypoxic environment. Hypoxia-primed epicardium activation induces a progenitor profile and shows multilineage differentiation ability (stromal and smooth muscle lineages) and the upregulation endothelial lineage markers, similarly to the perinatal epicardial profile. This figure was generated using Adobe Illustrator CC 2019 Version 23.0.2 (https://www.adobe.com/fr/).

We demonstrated previously that PW1 is broadly expressed during embryonic development and maintained in multiple adult progenitor niches^36–38^. We find a similar pattern of expression occurs in the heart with an initial widespread expression until early postnatal life and then restricted to the epicardium in the adult (Fig. 6).

Although, definitive models for cell fate tracing are needed to clearly address whether hypoxia induces an endothelial cell fate in Gp38^+^PW1^+^ epicardial cells, our single cell transcriptional data provides a static picture of cells at the time of isolation, it nonetheless provides an unbiased approach that strongly supports a model in which the multipotent epicardium contributes to cells with a stromal profile, and hypoxia upregulates the expression of several endothelial lineage-associated genes in the adult epicardium, similar to what occurs during perinatal cardiac development. Hypoxia has been shown to decrease fibrotic scarring coupled with functional recovery of the heart following ischemia^30^ and we demonstrated previously that PW1 stromal cells contribute to fibrosis following myocardial infarction^39^. Here, our single cell transcriptome analysis was able to detect different developmental state of epicardial and subepicardial-derived cells. Gp38^+^PW1^+^ epicardial cells show a strong lineage relationship between epicardial cells and PDGFRα^+^ cells (subepicardial and stromal cells) and to a lesser extent, but clearly more robust after hypoxia exposure, between epicardium and endothelial cells. The presence of a distinct subset of epicardial cells with endothelial gene expression after hypoxia exposure is analogous to the developing epicardium profile, indicating the potential of adult resident epicardial cells to up-regulate the expression of endothelial genes. However, we cannot confirm the complete endothelial differentiation, presumably reflecting the absence of proper/specific endothelial culture conditions used in our expansion assays. We reported previously that cardiac PW1^+^ stromal cells can be purified based upon the expression of αV-integrin and that pharmacological inhibition of αV-integrin results in a potent inhibition of fibrosis in response to ischemia coupled with a reduced infarct size^40^. Whether hypoxia and αV-integrin action share a common signaling pathway endpoint will be of interest to pursue. These results provide a cellular basis by which physiological hypoxia promotes angiogenesis. The precise pathways acting in response to hypoxia likely involve well characterized cell stress pathways that are also regulated by *Pw1*, such as TNFα-NFκB signaling in cell growth and survival^61^ and p53 signaling in apoptosis^60,86^, as well as the glucose metabolism regulation^65^.

This study also provides a novel strategy to purify and culture Gp38^+^PW1^+^ epicardial cells that will prove invaluable to further dissect mechanisms underlying the role of epicardial cells during injury and repair. We demonstrate that perinatal-derived Gp38^+^PW1^+^ epicardial cells have clonogenic capacity and can, self-renew as well as give rise to multiple lineages at the single cell level. Specifically, these cells express proteins associated with subepicardial and stromal cells (PDGFRα^+^), smooth muscle cells (αSMA^+^SM22α^+^) and Flk1, which is associated with immature endothelial cells. We demonstrate that cell fate potential is dependent upon oxygen levels and further show that epicardial cells isolated from the adult heart are markedly potentiated to expand in vitro following hypoxic priming in vivo. Taken together, this study demonstrates that resident Gp38^+^PW1^+^ epicardial cells retain progenitor competence in the adult mammalian heart and that hypoxia is critical to promote progenitor expansion and multi-lineage differentiation, including the up-regulation of an immature endothelial-like cell program. These findings serve as a foundation to design therapeutic approaches to promote heart revascularization and provide an invaluable in vitro model, which has been lacking to date for further studies.

## Methods

A detailed methods section is provided as online Supplementary Material.

### Mice

All animal procedures were approved by our institutional research committee (CEEA34 and French ministry of research) and conformed to the animal care guideline in Directive 2010/63/EU European Parliament. All animals received humane care in compliance with the “Principles of Laboratory Animal Care” formulated by the National Society for Medical Research, the “Guide for the Care and Use of Laboratory Animals” prepared by the Institute of Laboratory Animal Resources and published by the National Institutes of Health (NIH Publication No. 86-23, revised 1996), and adhered to the ARRIVE guidelines. All sacrifices were performed under deep anesthesia with isoflurane sufficient to minimize animal suffering followed by cervical dislocation.

C57BL/6J mice from Janvier and *PW1*^*IRESnLacZ*^ (*PW1*^*nlacZ*^) transgenic reporter mice^36^ were used.

### In vivo hypoxia

A cage connected to nitrogen and oxygen gas (ProOxP360, Biospherix) was used for the in vivo hypoxia with controlled atmosphere by silica gel (Carlo Erba) and soda lime (Intersurgical). Mice were exposed to hypoxia (10% O_2_) during 2 weeks.

### Cardiac cell suspension

Heart fragments were incubated at 37 °C with enzymatic solution (Collagenase II and DNase I) for serial rounds of digestion.

### Flow cytometry and cell sorting

Cell suspensions were stained with a panel of surface antibodies, C_12_FDG to detect ß-galactosidase activity and viability dye before flow cytometry analysis. The list of antibodies for flow cytometry is provided in Supplemental Material.

### Primary cell culture and cell immunofluorescence staining

Sorted epicardial cells (Gp38^+^PW1^+^) were plated in amplification medium under normoxia (21% O_2_) or acute hypoxia (1% O_2_) for 8 days. Cells were fixed, permeabilized and blocked. Primary antibodies were incubated followed by the adequate secondary antibodies and counterstained with DAPI. The list of antibodies for cell culture is provided in Supplementary Material.

### Histological processing and tissue immunofluorescence staining

Hearts were frozen, embedded in OCT and cut in four distinct coronal regions. Sections were fixed, permeabilized and blocked. Primary antibodies were incubated followed by the adequate secondary antibodies and counterstained with DAPI. The list of antibodies for histology is provided in Supplementary Material.

### X-gal staining

ß-galactosidase activity (LacZ) was detected in heart cryosections as described previously^87–89^.

### Pimonidazole injection and detection

Pimonidazole was injected intraperitonealy in adult mice (90 min incubation) and injected subcutaneously in newborn mice (3 h incubation) before heart collection. Hearts were frozen and pimonidazole was detected with MAb1 (Hydroxyprobe), according to the manufacturer’s instructions.

### Multiplex qPCR (bulk and single cell)

Cells were sorted directly into RT-STA reaction mix (CellsDirect One-Step qRTPCR Kit) and 0.2 × specific TaqMan Assay mix. Taqman assays are listed in Supplemental Material. Multiplex qPCR was preformed using the microfluidics Biomark HD system (Fluidigm) as previously described^90^ for the same TaqMan Assay panel in Supplementary Material.

### Bioinformatic analysis

Gene expression raw data (Biomark, Fluidigm) of both bulk and single cell were normalized with Gapdh housekeeping gene, and ‘heatmap’, ‘Rphenograph’ and ‘diffusion map’ were created using R packages^91^.

### Quantifications and statistical analyses

Histological images were analyzed with Icy software^92^. Graphs and statistics were performed with Prism software (GraphPad). The line in the box plots represents the median value. For the statistical comparison, two groups were tested by Mann–Whitney U test and for more than two groups one-way analysis of variance (ANOVA) was used. A value of p < 0.05 was considered significant. Biological replicates represent independent experiments.

## Supplementary Information

Supplementary Information 1.

Supplementary Information 2.
