# Supplementary figures and images for: Hypoxia promotes a perinatal-like progenitor state in the adult murine epicardium
Source: Sci Rep. 2022 Jun 3;12:9250. doi: 10.1038/s41598-022-13107-2 (PMC9166725; doi:10.1038/s41598-022-13107-2)

**A**

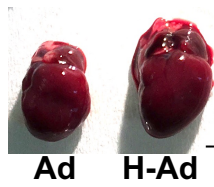

# B

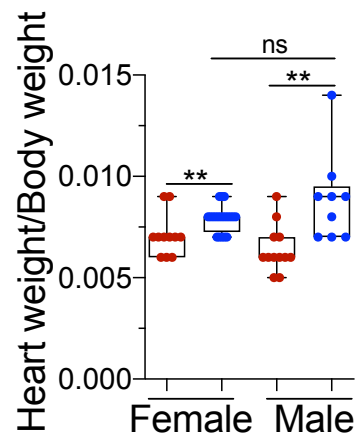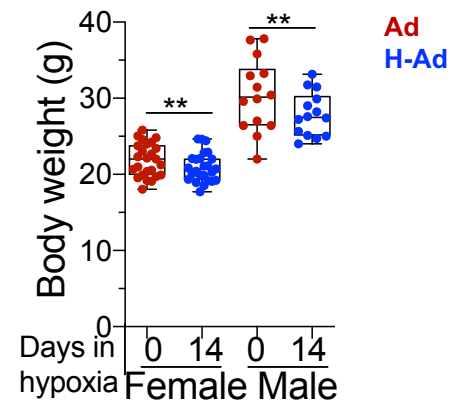

**C**

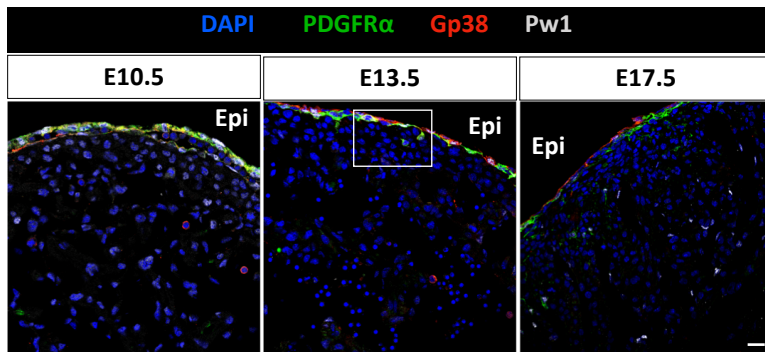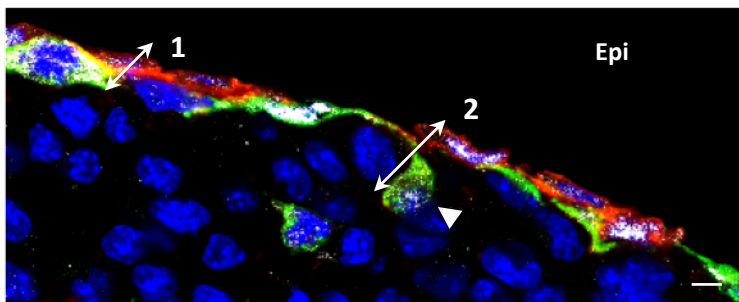

**D**

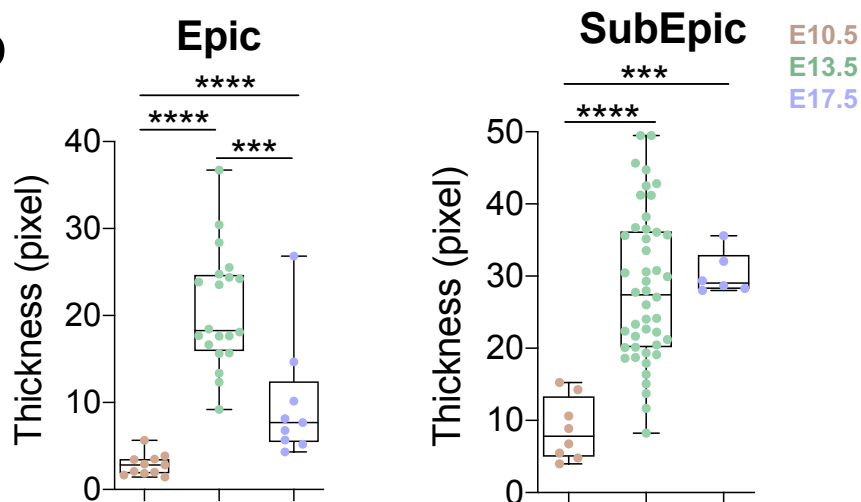

## A

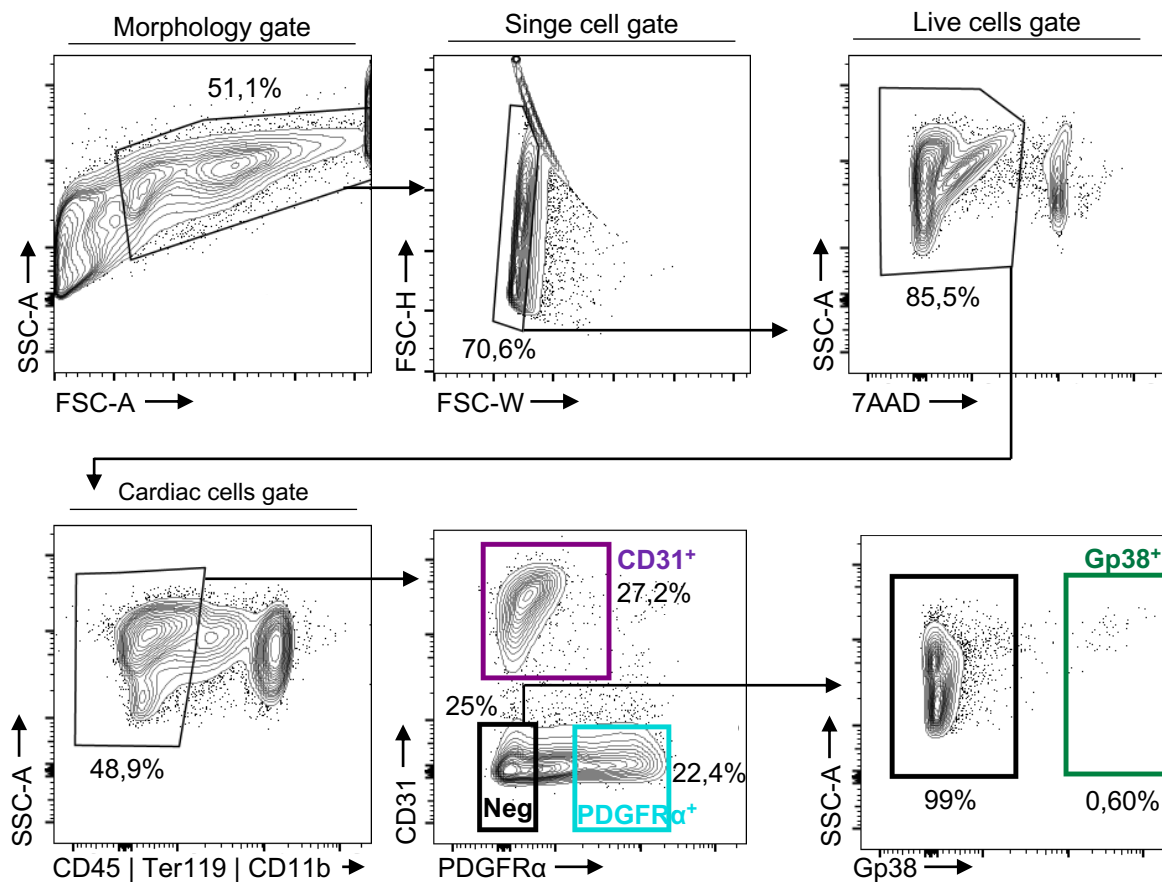

## B

Live cells

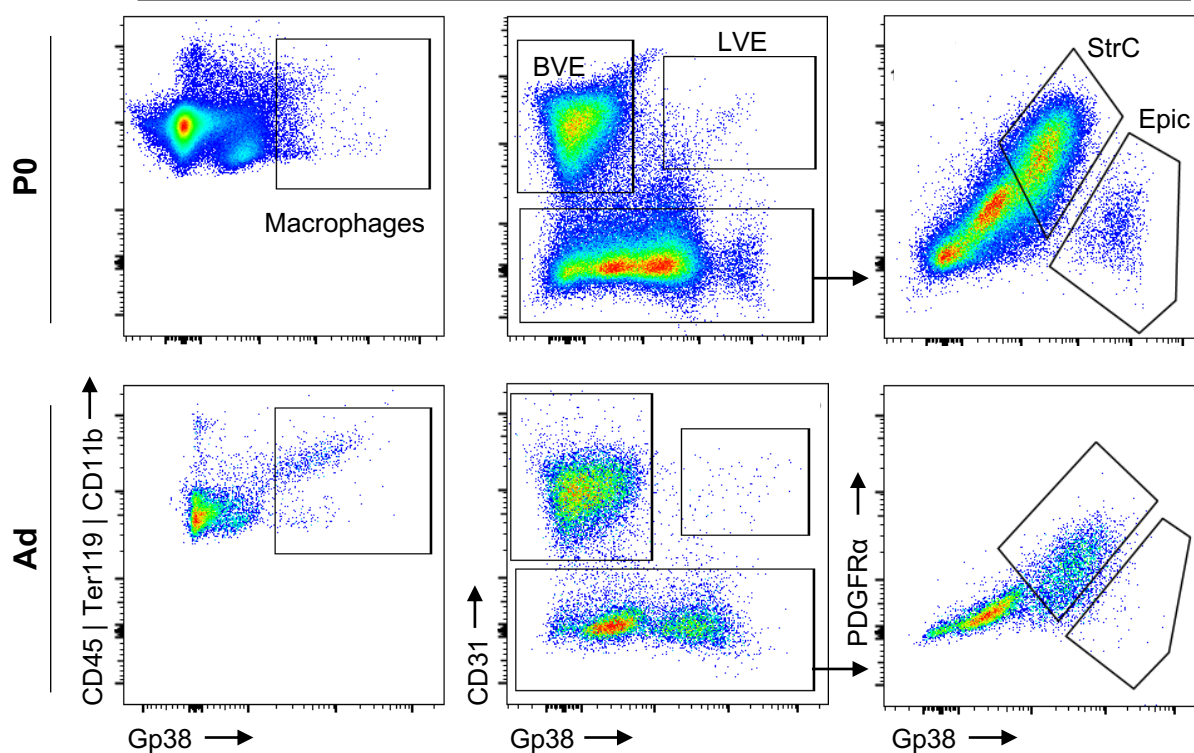

## A

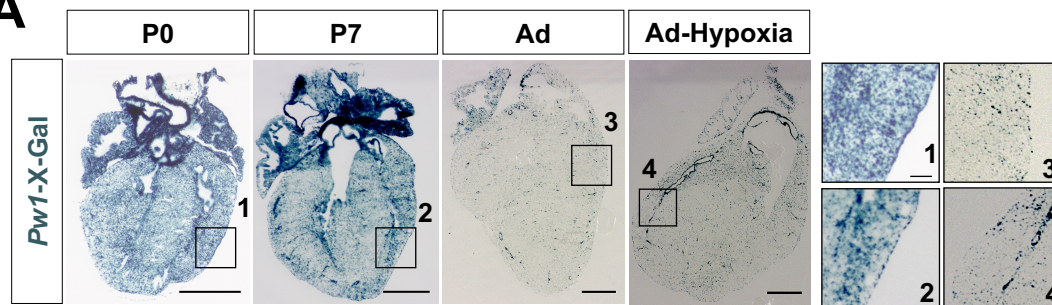

## B

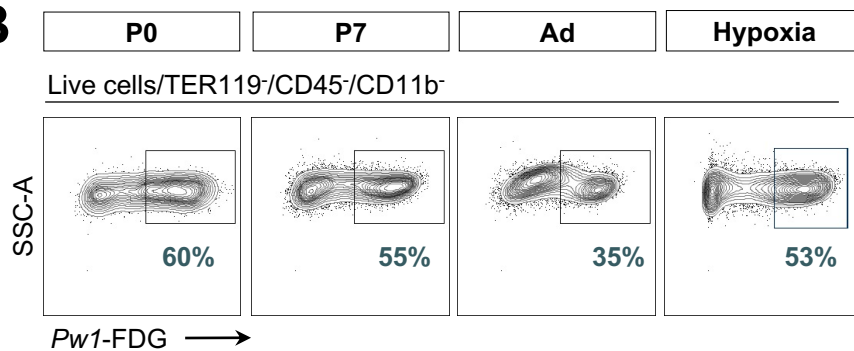

## C

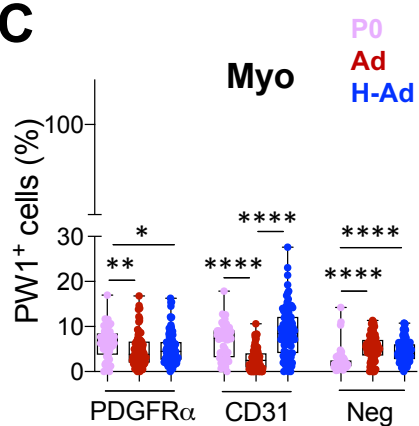

## D

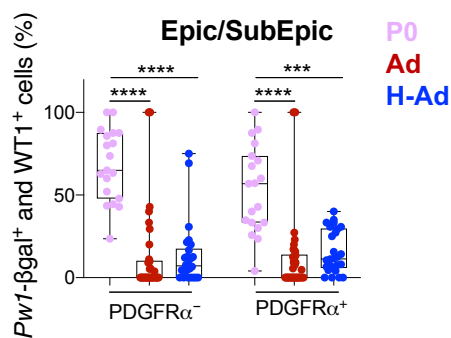

## E

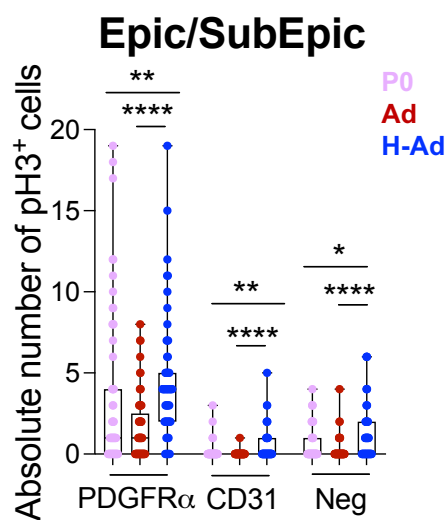

## F

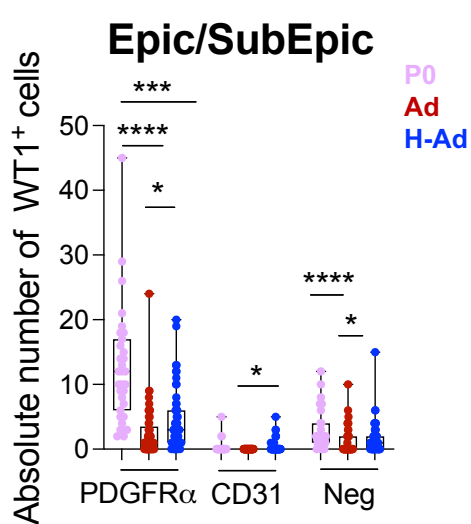

**A**

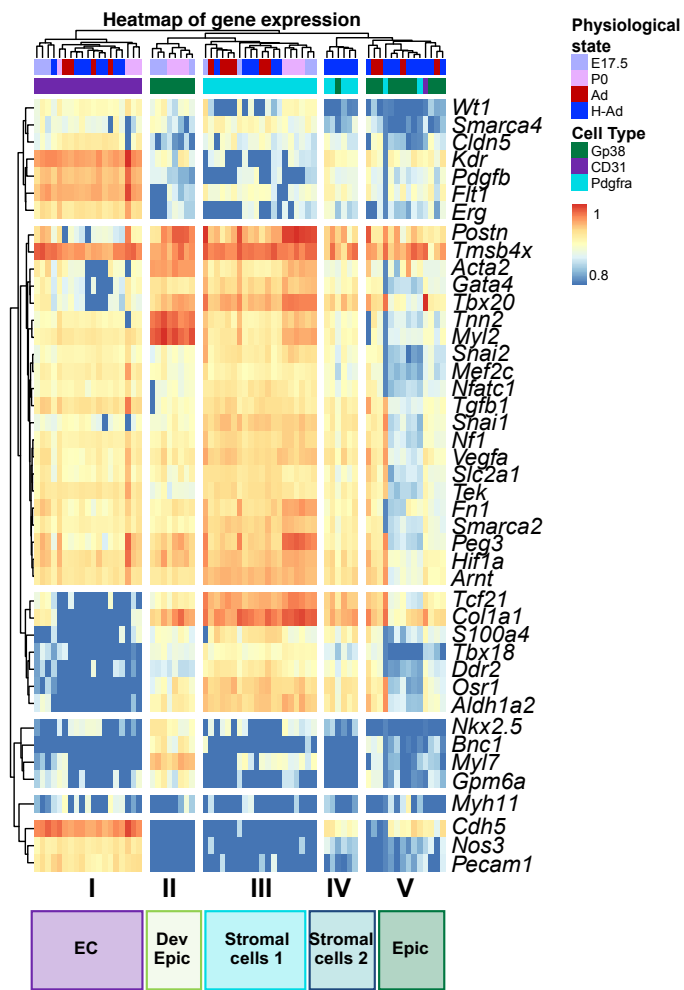

**B**

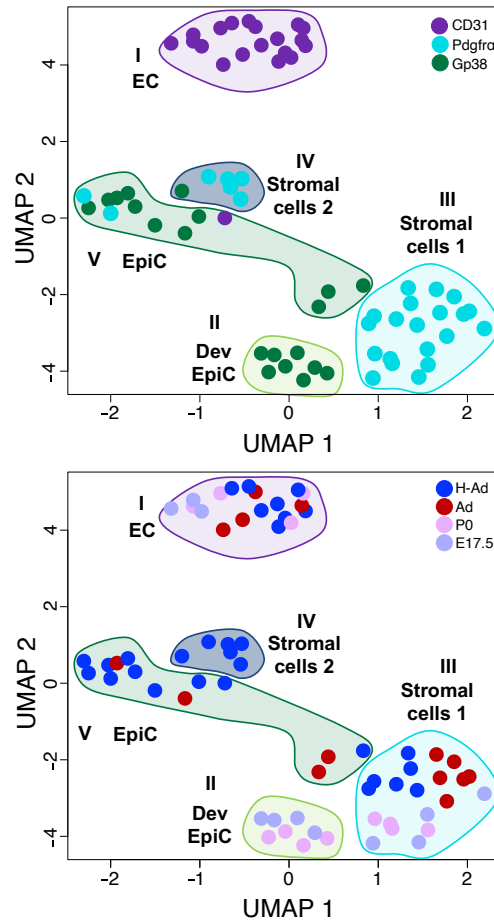

**C**

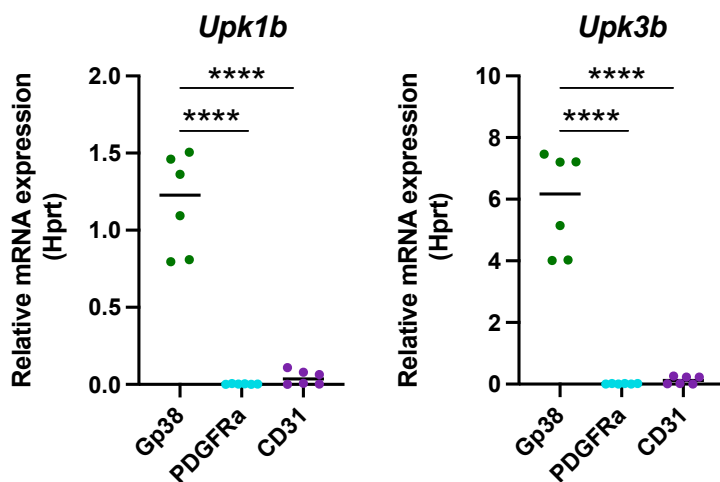

**Physiological state**

- E17.5
- P0
- Ad

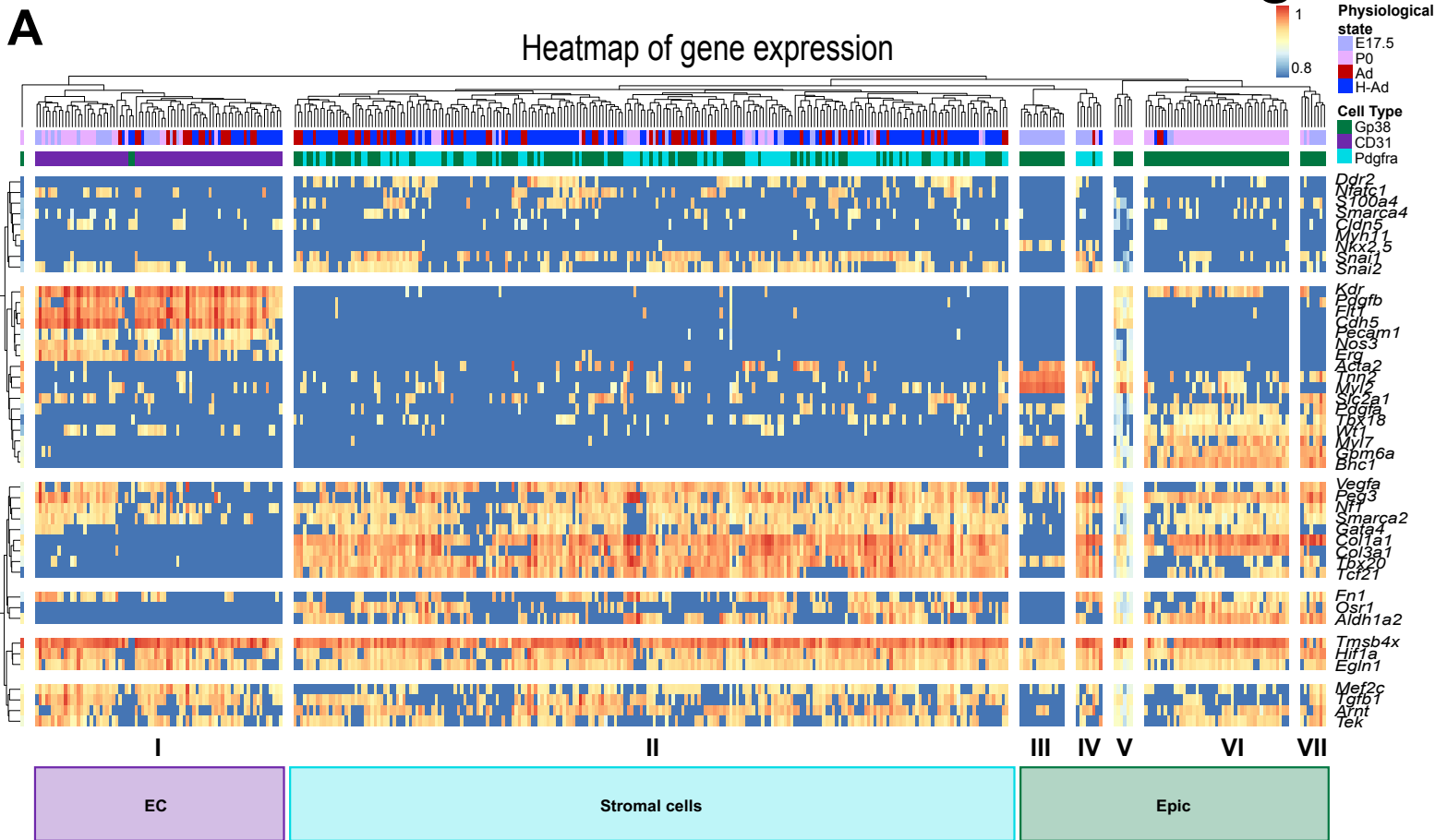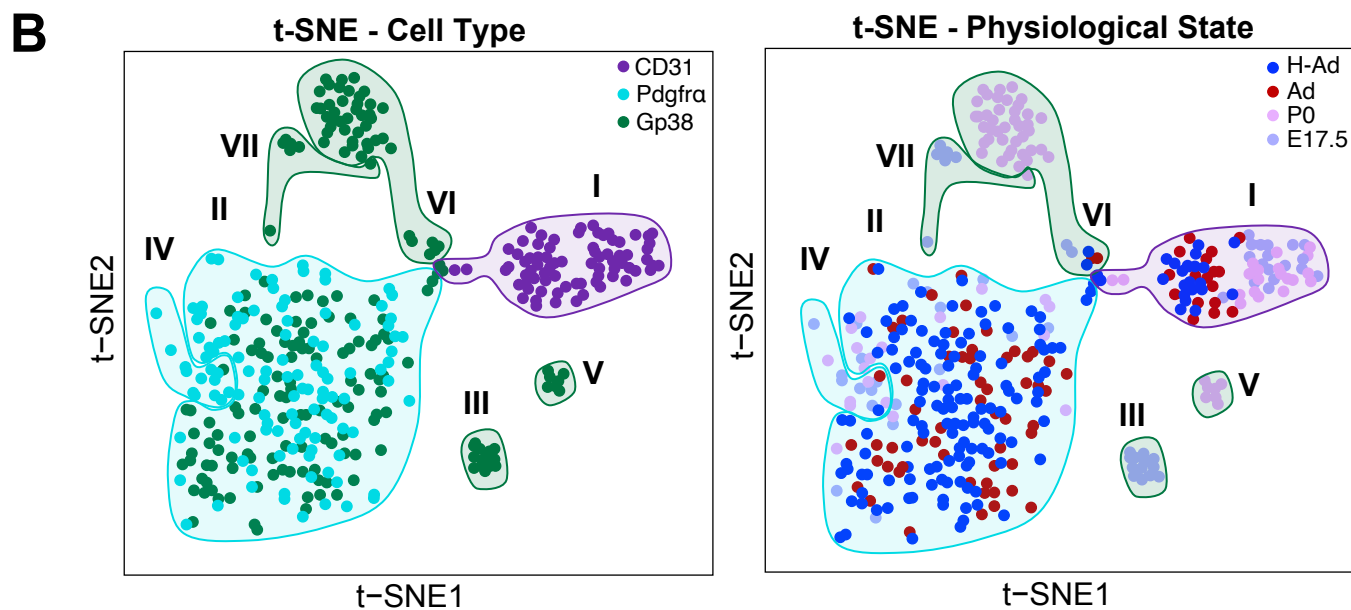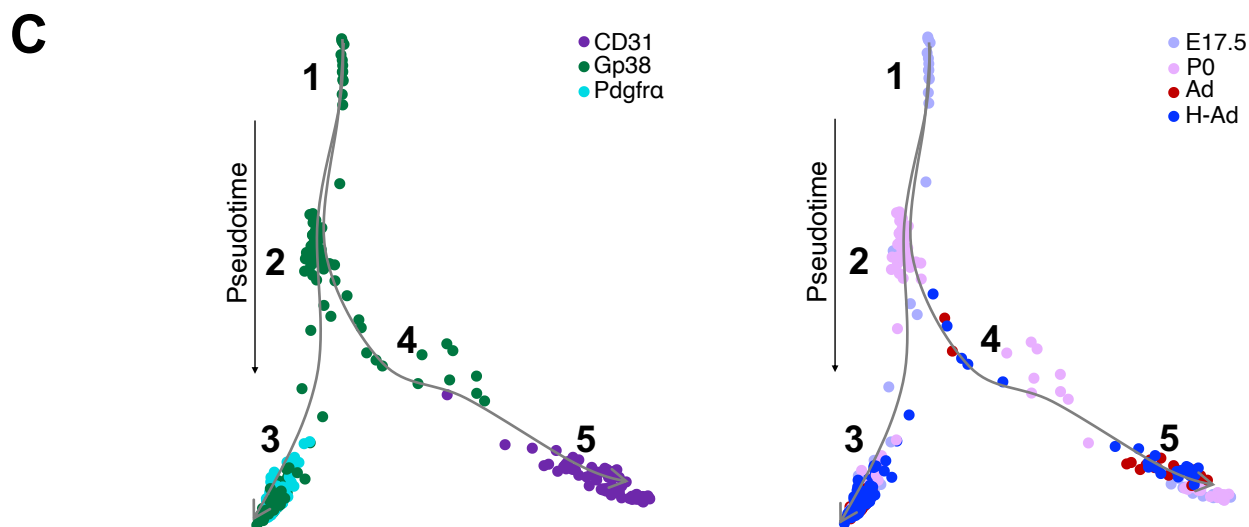

# Fig S VI

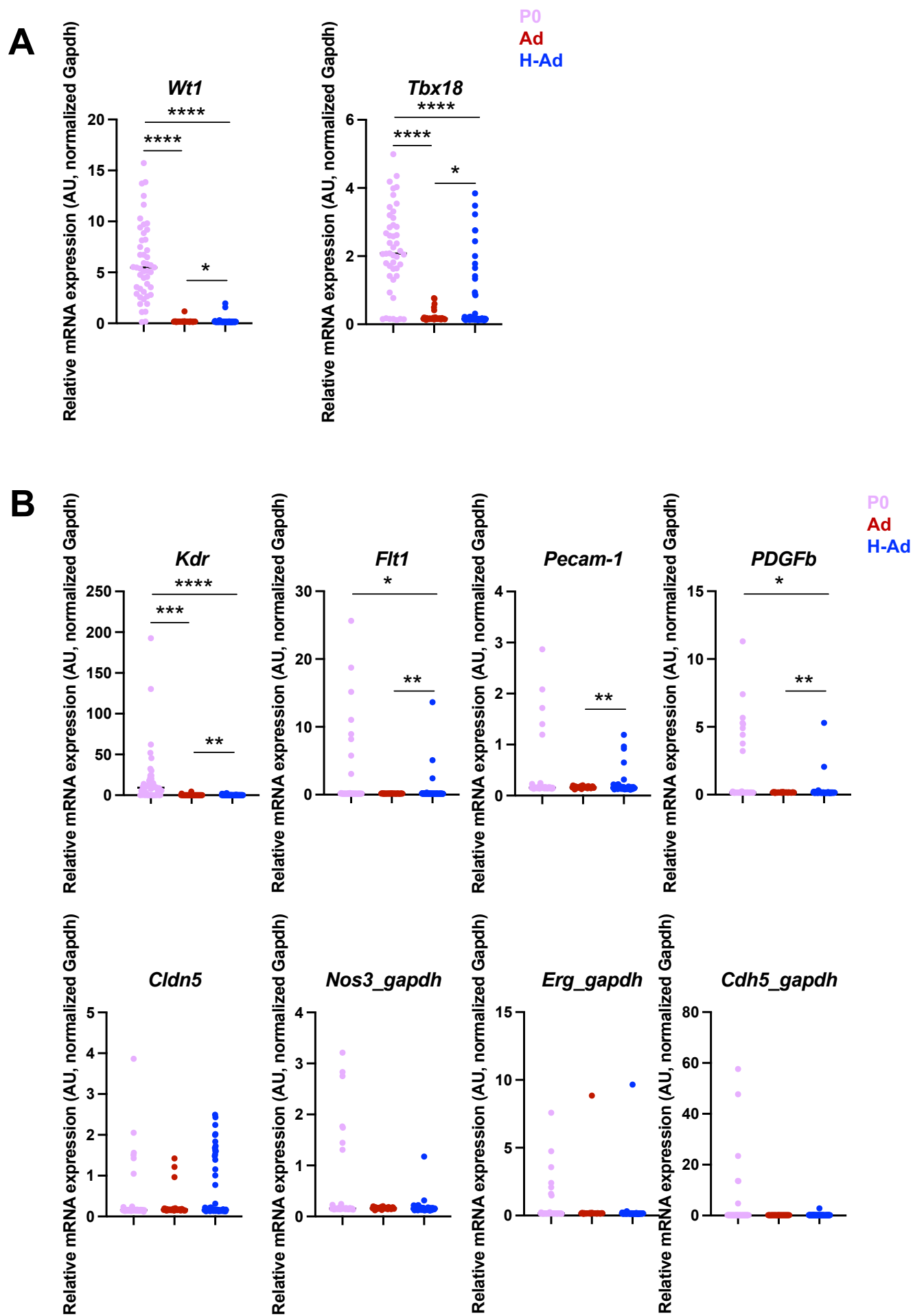

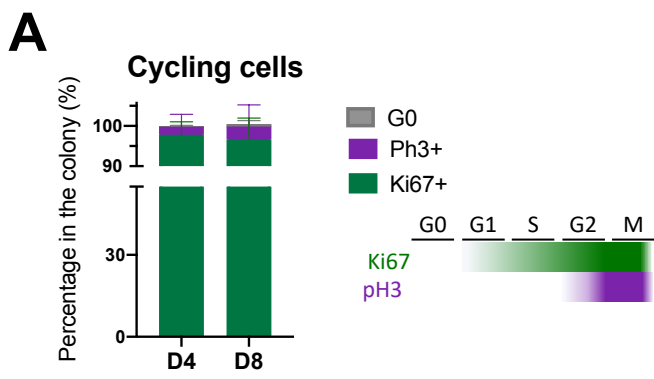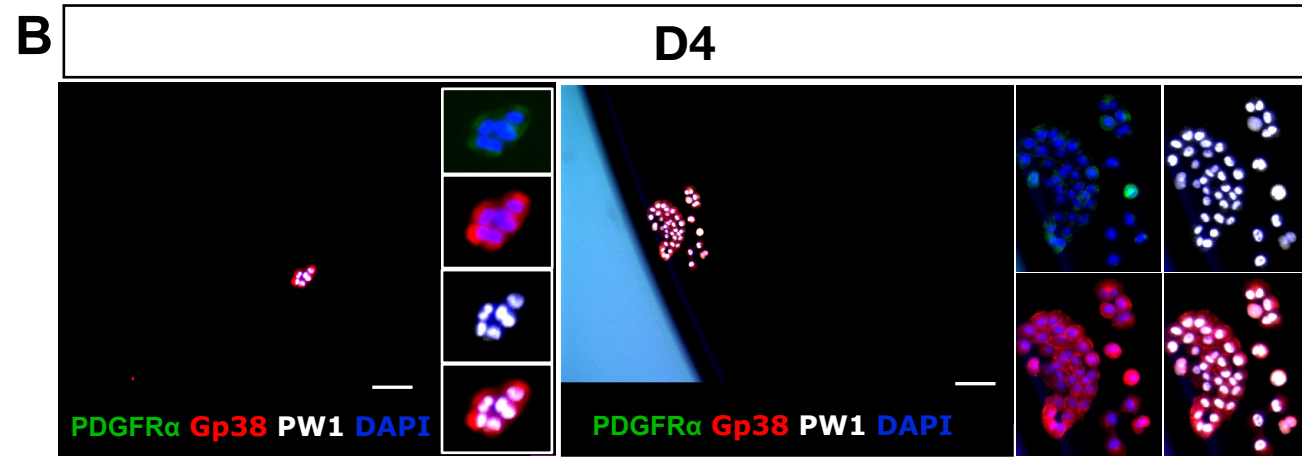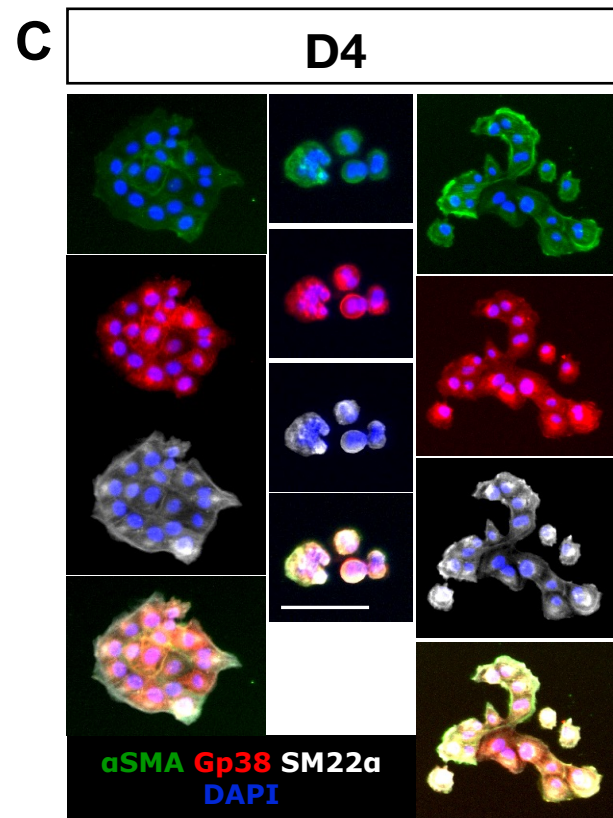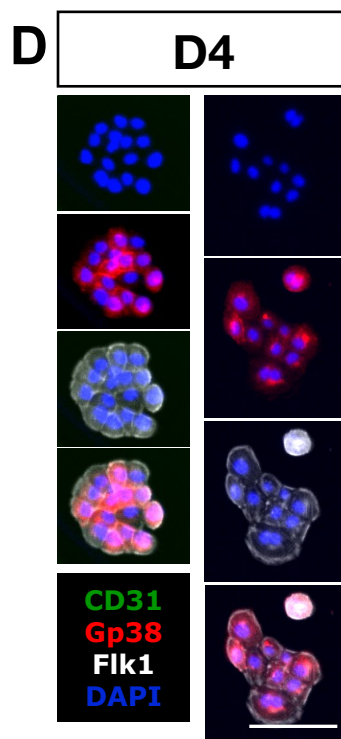

**Fig S VIII**

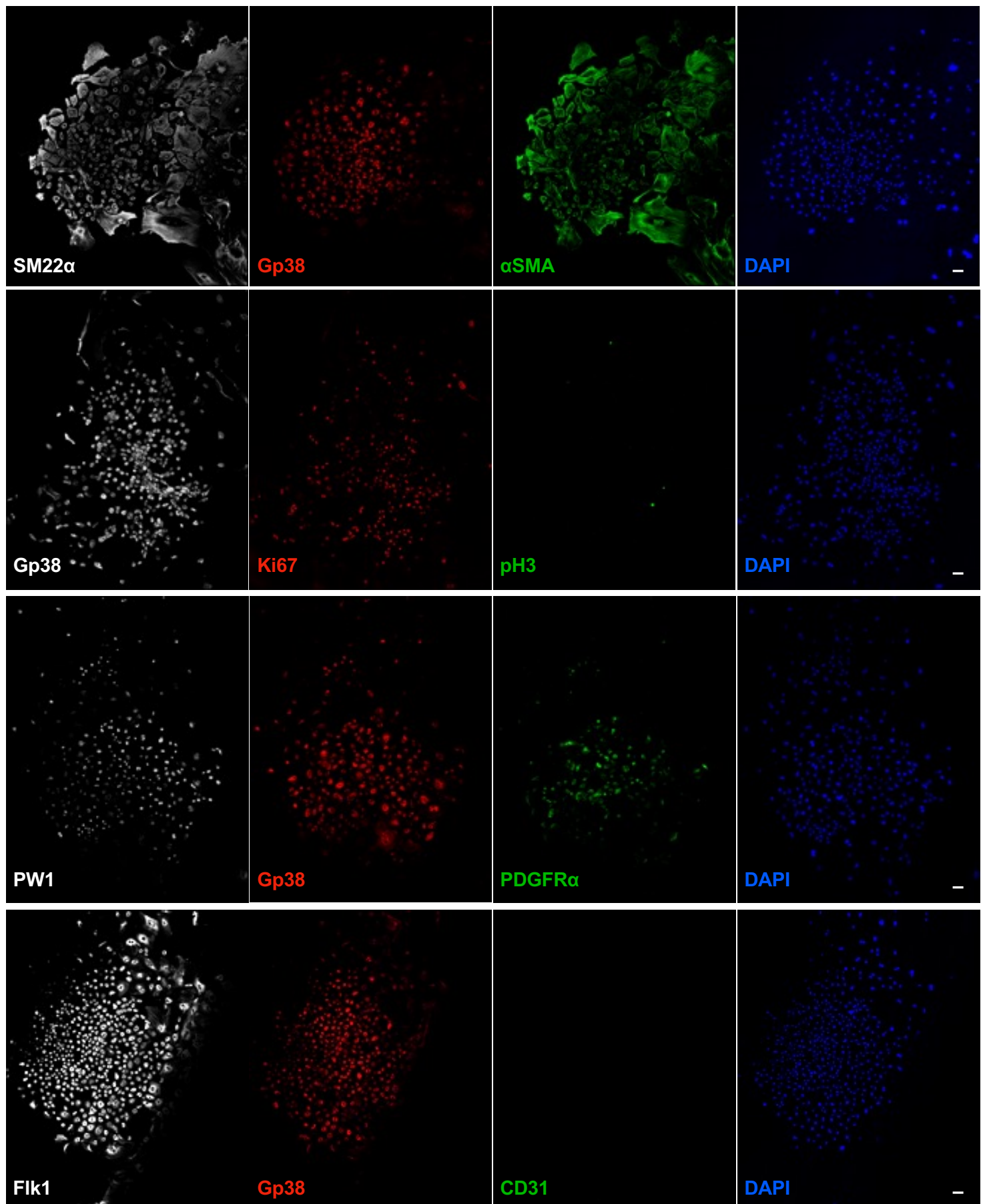

Supplement: Supplementary file 1 — Supplementary Information 1. [file 41598_2022_13107_MOESM1_ESM.pdf]
