# Supplementary material for: Hypoxia promotes a perinatal-like progenitor state in the adult murine epicardium
Source: Sci Rep. 2022 Jun 3;12:9250. doi: 10.1038/s41598-022-13107-2 (PMC9166725; doi:10.1038/s41598-022-13107-2)
Supplement: Supplementary file 2 — Supplementary Information 2. [file 41598_2022_13107_MOESM2_ESM.docx]

**SUPPLEMENTAL MATERIAL**

**Hypoxia promotes a perinatal-like progenitor state in the adult murine epicardium**

Angeliqua Sayed, PhD^1^; Szimonetta Turoczi, Ms^1^; Francisca Soares-da-Silva, PhD^2^; Giovanna Marazzi, MD^1^; Jean-Sébastien Hulot, MD, PhD^1,3^; David Sassoon, PhD^1^ and Mariana Valente, PhD^1^

Affiliations:

^1^Université de Paris, INSERM- U970, PARCC, F-75006 Paris, France;

^2^Lymphocytes and Immunity Unit, Immunology Department, Institut National de la Santé et de la Recherche Médicale U1223, Institut Pasteur, Paris, France;

^3^CIC1418 and DMU CARTE, AP-HP, Hôpital Européen Georges-Pompidou, F-75015, Paris, France.

Corresponding author:

Dr. David Sassoon

Email: david.a.sassoon@gmail.com

**Extended Methods**

**Mice**

C57BL/6J mice were purchased from Janvier and *PW1IRESnLacZ* (*PW1nlacZ*) transgenic reporter mice has been previously described^21^. Primed-pregnant females, 8-12 weeks-old mice (adult females or males) and 56 weeks-old mice (aged-adult females or males) were used. Timed pregnancies were generated after overnight mating. The following morning, females with vaginal plug were considered to be at E0.5. Both embryonic and postnatal hearts were used. Mice body weight and heart weight were taken from all conditions.

All animal procedures were approved by our institutional research committee (CEEA34 and French ministry of research) and followed the animal care guideline in Directive 2010/63/EU European Parliament.

***In vivo* hypoxia experiment**

An animal cage enclosure (A-Chamber, Biospherix) was used for the *in vivo* hypoxic experiment. The chamber was connected to nitrogen and oxygen gas controller (ProOxP360, Biospherix) in order to monitor the atmosphere condition inside the chamber, and had an oxygen sensor, an internal circulation fan and passive circulation. Silica gel (Carlo Erba) was used to absorb the humidity and soda lime (Intersurgical) was used to remove the excess of carbon dioxide in the chamber atmosphere. A portable oxygen gas sensor was placed inside the chamber to verify the stability of the oxygen level over time. *PW1nlacZ* or C57BL/6J adult mice were exposed to hypoxia (10% O_2_) during 2 weeks and compared with mice kept in normoxic (20.9% O_2_). Mice were then euthanized and hearts harvested for histological analyzes or cell dissociation for *in vitro* and qPCR analyses, according to the procedures described below. We observed chronic hypoxia induced heart enlargement and a slight decrease in the absolute body weight (Figure III-A-B in the Supplement), reflecting a reduction of fat mass described by others^26^. No significant differences were observed between males and females heart/body weight ratio (Figure III-A-B in the Supplement) and thus both sexes were used for our analyses.

**Cardiac cell suspension**

Hearts were collected and washed with cold HBSS with Ca^2+^Mg^2+^ (HBSS+/+, ThermoFisher) supplemented with 1% FBS (GE Healthcare Life Sciences, 1% FBS-HBSS+/+). Ventricles were cut in small pieces (1mm^3^), washed in 1%FBS-HBSS+/+ and incubated at 37°C for 15 minutes in 2mL of enzymatic solution (500U/mL of Collagenase II (Worthington) and 32 U/mL of DNase I (Sigma-Aldrich)). After each round of digestion, samples were resuspended and the remaining non-digested tissue was let precipitate. The supernatant was transferred to an equivalent volume of 10%FBS-HBSS+/+ and kept on ice. To the remaining non-digested tissue, 2mL of new enzymatic solution was added and the digestion procedure was repeated until no more tissue fragments were observed. After digestion, the cell suspension was filtered with a 70μm mesh strainer (Corning) and centrifuged 10 minutes, 290 rcf at 4°C. For the adult epicardial cells isolation, we performed an additional step of sample enrichment for the epicardial layer, which consists in removing the muscle of the inner part of the ventricle wall before the digestion procedure.

**Flow cytometry and cell sorting**

Cell suspensions were stained for 20 minutes at 4°C in the dark with the following antibodies: TER119 – BUV395 (TER-119, BD Biosciences), CD45 – BUV395 (30-F11, BD Biosciences), CD11b – BUV395 (M1/70, BD Biosciences), Gp38 – PE-Cy7 (eBio8.1.1, Invitrogen), PDGFRα - PE (APA5, BD Biosciences) and CD31 – BUV737 (53-2.1, BD Biosciences). After incubation, cells were centrifuged 5 minutes, 290rcf at 4°C and resuspended in the washing solution 1%FBS-HBSS+/+. For *Pw1LacZ* detection, 5-Dodecanoylaminofluorescein Di-β-D-Galactopyranoside (C_12_FDG, Thermofisher) staining was performed after the surface membrane staining. Samples were incubated in 600μL of washing solution and 20μL of C_12_FDG (2mM) at 37°C for 1h, centrifuged (5 minutes, 290 rcf at 4°C) and washed.

Before acquisition/sorting stained-cell suspensions were filtered with 70μm mesh (Fisherbrand™) and 7AAD (BD Biosciences) was used to exclude dead cells.

Stained cells were analyzed following the gating strategy described in the Figure II in the Supplement on a BD LSR Fortessa and BD Fortessa X20 (BD Bioscience) and were sorted in a BD FACSAria II (BD Bioscience), according to the guidelines for the use of flow cytometry and cell sorting^34^. The purity of sorted Gp38^+^ cells was verified for the absence of the surface markers CD31, PDGFRα, CD11b, CD45 and Ter119 prior to plating (Figure 5A). Data were analyzed with FlowJo software (BD Bioscience, v.10.6.1).

**Primary cell culture and cell immunofluorescence staining**

For limiting dilution analysis, sorted epicardial cells (Gp38^+^PW1^+^) were plated in non-coated plates at different densities (1000, 500, 100, 50, 10, 1cells/cm^2^) in amplification medium - Dulbecco’s modified Eagle medium (DMEM, Gibco) high glucose (4.5g/l) supplemented with 20% of heat-inactivated fetal bovine serum (FBS, Hyclone), 10% of horse serum (HS, Gibco), 1% (v/v) penicillin-streptomycin (Gibco), 1% (v/v) L-Glutamine (Gibco), 1% (v/v) sodium pyruvate (Gibco) and 1% (v/v) HEPES (Gibco). Cells were cultured under normoxia (21 % O_2_) or acute hypoxia (1% O_2_) by using a humidified hypoxic incubator chamber (Billups Rothenberg, Brincubator). The oxygen levels were continuously monitored with a portable oxygen gas sensor (Indsci). At day 2, additional amplification medium was added to allow the cells to settle down, and from day 4 onwards, the amplification medium was changed every two days. After 7-9 days, wells were assessed for the presence of epicardial colonies. Cell frequencies determined with extreme limiting dilution analysis (ELDA) software, from Walter and Eliza Hall Institute Bioinformatics Division – Institute of Medical Research, are presented as the number of positive wells and the number of total tested wells^35^ Brightfield images were taken with EVOS Epifluorescence microscope (Thermofisher).

For colony characterization, sorted cells were plated at 100 cells/cm^2^ culture dishes or 𝜇-slide 8 wells (Ibidi) and colonies were assessed at 8 days and markers of the different epicardial lineages were used.

Cells were fixed with 4% paraformaldehyde (PFA, Electron Microscopy Sciences) for 15 minutes, permeabilized with 0.5% Triton 100x for 10 minutes and blocked with 5% bovine serum albumin (BSA, Jackson ImmunoReasearch) for 1 hour. Primary antibodies were incubated overnight at 4°C with 40 rpm stirring. The following antibodies were used: sheep anti-mouse ki67 (R&D Systems), rabbit anti-mouse pH3 (Merck), rabbit anti-mouse PW1^36^, golden Syrian hamster anti-mouse Gp38 (Novus Biologicals), goat anti-mouse PDGFRα (R&D systems), mouse anti-mouse αSMA (Sigma), rabbit anti-mouse SM22α (abcam), rat anti-mouse CD31 (BD Pharmigen) and rabbit anti-mouse Flk1 (abcam). Cells were then incubated with the secondary antibodies for 1 hour at room temperature in the dark with 40 rpm stirring and counterstained with DAPI. Images were taken with EVOS Epifluorescence microscope (Thermofisher) with the objectives 10x and 20x or mosaic module from the entire colony with the confocal SP8 (Leica), objective 40x, frame average equal to three for each channel and image size of 1024x1024.

**Histological processing and tissue immunofluorescence staining**

Hearts were frozen in 2-propanol cooled by dry ice in OCT and stored at -80°C. Whole heart representation was assured by the collection of four equidistant (150-200μm of distance) coronal sections (8μm thickness). In a humid histological chamber, tissues were fixed in 2% PFA for 15 minutes at room temperature, permeabilized in 0.5% Triton 100x (Sigma) for 10 minutes and blocked with 5% BSA or M.O.M kit (Vector Laboratories) during 1 hour at room temperature. Tissue sections were stained with primary antibodies diluted in blocking solution overnight at 4°C followed by the adequate secondary antibodies diluted in blocking solution at room temperature in the dark during 1 hour. The following primary antbodies were used: sheep anti-mouse ki67 (R&D Systems), rabbit anti-mouse pH3 (Merck), rabbit anti-mouse PW1^36^, golden Syrian hamster anti-mouse Gp38 (Novus Biologicals), rabbit anti-mouse WT (abcam), goat anti-mouse PDGFRα (R&D systems), rat anti-mouse CD31 (BD Pharmigen), rabbit anti-mouse laminin (Sigma) and mouse antibody IgG1, MAb1 (Pimonidazole, Hypoxyprobe). Nuclei were counterstained with DAPI (Sigma-Aldrich) and the slides were mount with Fluoromount G (Southern Biotech). We performed a sequential immunostaining for antibodies with any protocol incompatibility or cross-reactivity. Images were taken with the confocal SP8 (Leica), objective 40x, frame average equal to three for each channel and image size of 1024x1024. For higher magnification images, the optimal zoom was determined by using the Nyquist sampling. Quantification was done in 3 hearts (n=3), with 4 sections by heart and 5 images were taken by section.

**X-gal staining**

LacZ was detected in heart cryosections as described previously^37-39^ with some modifications. Cryosections were placed in a highly humid chamber and fixed 5 min at 4°C with the following fixative solution: 2% formaldehyde (Electron Microscopy Sciences), 0.20% of Glutaral (Sigma-Aldrich), 5% NP40 (ThermoFisher) and 0.01% Nadeoxycholate (ThermoFisher) diluted in 1x PBS. Sections were washed and incubated with the following solution pre-warmed (at 35°C): 5mM potassium ferricyanide (III) (Sigma-Aldrich), 5mM potassium hexacyanoferrate (II) (Sigma-Aldrich), 2mM magnesium chloride (Sigma-Aldrich), 0.02% NP40 (ThermoFisher) 0.01% Nadeoxycholate (ThermoFisher) and 40mg/mL of Xgal (Roche) diluted in 1x PBS in sealed humid chamber at 35°C, overnight. The day after, sections were washed and kept in 1x PBS overnight, at 4°C. Slides were mount with Fluoromount G medium. Images were taken with Leica L2 (Leica).

**Pimonidazole injection and detection**

Pimonidazole is reductively active in hypoxic cells by the formation of covalent adducts with thiols groups in proteins, peptides and amino acids present in the hypoxic cells (partial pressure equal or below pO_2_ = 10 mmHg at 37°C).

Adult normoxic and hypoxic mice were intraperitoneal injected with 300 mg/kg of pimonidazole (Hypoxyprobe) and heart was harvested 90 minutes following the injection. Hypoxia-primed adult mice were kept in the hypoxia chamber after pimonidazole administration. Newborn mice were subcutaneous injected with 300 mg/kg of pimonidazole (Hypoxyprobe), placed in a warm pad for 3 hours followed by heart collection. For histological analyses, hearts were frozen and the pimonidazole was detected with MAb1 (Hydroxyprobe), according to the manufacturer’s instructions.

**Multiplex qPCR (bulk and single cell)**

100 cells/well or single cells were sorted directly into 96-well plates loaded with RT-STA reaction mix (CellsDirect One-Step qRTPCR Kit, Invitrogen; according to the manufacturer’s instructions) and 0.2x specific TaqMan Assay mix and stored at -80°C at least overnight. The following Taqman assays were used: *Peg3* - Mm01337379_m1; *Osr1* -Mm00726877_m1; *Wt1* - Mm01337048_m1; *Tbx18* - Mm00470177_m1; *Smarca4* - Mm01151944_m1; *Smarca2* - Mm00508992_m1; *Nf1* - Mm00812424_m1; *Tmsb4x* - Mm01129684_g1; *Gpm6a* - Mm00463812_m1; *Bnc1* - Mm01324337_m1; *Nfatc1* - Mm01265944_m1; *Pecam1* - Mm01242576_m1; *Erg* - Mm01214244_m1; *Slc2a1* - Mm00441480_m1; *Nkx2-5* - Mm01309813_s1; *Tnnt2* - Mm01290256_m1; *Myl7* - Mm00491655_m1, *Tbx20* - Mm00451515_m1; Col3a1 - Mm00802300_m1; *Postn* - Mm01284919_m1; *Ddr2* - Mm00445615_m1; *Tcf21* - Mm00448961_m1; *Aldh1a2* - Mm00501306_m1; *Tgfb1* - Mm01178820_m1; *Snai1* - Mm00441533_g1; *Snai2* -Mm00441531_m1; *Tek* - Mm00443243_m1; *Kdr* - Mm01222421_m1; *Flt1* - Mm00438980_m1; *Cdh5* - Mm00486938_m1; *Cldn5* - Mm00727012_s1; *Pdgfb* - Mm00440677_m1; *Vegfa* - Mm00437306_m1; *Nos3* - Mm00435217_m1; *Hif1a* - Mm00468869_m1; *Arnt* - Mm00507836_m1; *Egln1* - Mm00459770_m1; *Acta2* - Mm01546133_m1; *Myh11* - Mm00443013_m1; *Myl2* - Mm00440384_m1; *Gata4* - Mm00484689_m1; *Mef2c* - Mm01340842_m1; *Col1a1* - Mm00801666_g1, *Fn1* - Mm01256744_m1; *S100a4* - Mm00803372_g1; *Pdgfra* - Mm00440701_m1; *HPRT* - Mm03024075_m1; *Gapdh* - Mm99999915_g1; *Actb* - Mm02619580_g1.

For single cell experiments, a control well with 100 cells was also sorted. Pre-amplified cDNA was obtained according to the manufacturer’s procedure (bulk-18 cycles and single cell-20 cycles) and diluted 1/5 in Tris-lowEDTA buffer (Sigma). Multiplex qPCR was preformed using the microfluidics Biomark HD system (Fluidigm) for 40 cycles as previously described^40^ for the same TaqMan Assay panel.

**Supplemental Table 1**

**List of genes specific of each cardiac lineage selected for the transcriptome analysis by multiplex qPCR (bulk and single cell).**

| **Gene symbol** | **Gene alias** | **Gene ID** | **Cell lineage association** |
| --- | --- | --- | --- |
| *Peg3* | Pw1 | 18616 | Imprinted  Stem cells |
| *Wt1* | Wt-1 | 22431 | Epicardial cells  Epicardial  derived cells |
| *Tbx18* | 2810012F10Rik | 76365 |  |
| *Aldh1a2* | Raldh1 | 19378 |  |
| *Smarca4* | Brg1; BAF190A | 20586 |  |
| *Smarca2* | brm; Snf2l2; brahma | 67155 |  |
| *Gpm6a* | M6A; Gpm6 | 234267 |  |
| *Bnc1* | Bnc; AI047752; AW546376 | 12173 |  |
| *Tgfb1* | Tgfb-1; TGFbeta1; TGF-beta1 | 21803 | Epicardial derived cells |
| *Nf1* | Dsk9; Nf-1 | 18015 |  |
| *Snai1* | Sna1; Snail; Snail1 | 20613 |  |
| *Snai2* | Slug; Slugh; Snail2 | 20583 |  |
| *Tmsb4x* | Tb4; Ptmb4; Tbeta4 | 19241 |  |
| *Nfatc1* | NFAT2; NFATc | 18018 |  |
| *Tek* | Hyk; STK1; Tie2; Tie-2 | 21687 | Endothelial cells |
| *Kdr* | Flk1; Krd-1; VEGFR-2; sVEGFR-2 | 16542 |  |
| *Flt1* | Flt-1; sFlt1; VEGFR1; VEGFR-1 | 14254 |  |
| *Pecam1* | Cd31; Pecam | 18613 |  |
| *Erg* | D030036I24Rik | 13876 |  |
| *Cdh5* | Vec; VECD; Cd144; VE-Cad | 12562 |  |
| *Cldn5* | MBEC1; Tmvcf; AI854493 | 12741 |  |
| *Pdgfb* | PDGF-2; PDGF-B | 18591 |  |
| *Vegfa* | Vpf; Vegf | 22339 |  |
| *Nos3* | eNOS; Nos-3; ecNOS | 18127 |  |
| *Acta2* | Actvs; a-SMA; SMAalpha; SMalphaA; alphaSMA | 11475 | Smooth muscle cells |
| *Myh11* | SM1; SM2; smMHC | 17880 |  |
| *Nkx2-5* | Csx; Nkx2.5; tinman; Nkx-2.5 | 18091 | Cardiomyocytes |
| *Tnnt2* | Tnt; cTnT | 21956 |  |
| *Myl2* | MLC-2; Mlc2v; MLC-2v | 17906 |  |
| *Myl7* | MLC2a; MYL2A; Mylc2a; MLC-2alpha | 17898 |  |
| *Gata4* | Gata-4 | 14463 | Cardiomyocytes  Stromal cells |
| *Tbx20* | Tbx12; AL022859 | 57246 |  |
| *Mef2c* | Mef2; AV011172 | 17260 |  |
| *Col1a1* | Cola1; Mov13; Cola-1; Mov-13; Col1a-1 | 12842 | Stromal cells |
| *Col3a1* | Tsk2; Tsk-2; Col3a-1 | 12825 |  |
| *Postn* | PN; PLF; Osf2; OSF-2 | 50706 |  |
| *Osr1* | Osr; Odd1 | 23967 |  |
| *Fn1* | Fn; Fn-1; E330027I09 | 14268 |  |
| *S100a4* | 2a; 18A2; Capl; FSp1; Mts1 | 20198 |  |
| *Ddr2* | Ntrkr3; tyro10; AW495251 | 18214 |  |
| *Tcf21* | epc; Pod1; Pod-1; bHLHa23; epicardin | 21412 |  |
| *Pdgfa* | PDGF-1 | 18590 |  |
| *Slc2a1* | Glut1; Glut-1 | 20525 | Metabolism |
| *Hif1a* | HIF1alpha; HIF1-alpha; HIF-1-alpha | 15251 |  |
| *Arnt* | Drnt; Hif1b; ESTM42 | 11863 |  |
| *Egln1* | Phd2; HPH-2; ORF13; SM-20; C1orf12; HIF-PH2 | 112405 |  |
| *Hprt* | HPGRT; Hprt1; C81579 | 15452 | Housekeeping |
| *Gapdh* | Gapd | 14433 |  |
| *Actb* | Actx; beta-actin; E430023M04Rik | 11461 |  |

**Supplement Figure Legends**

**Figure S I. Validation of the *in vivo* hypoxia model and embryonic dynamic of the epicardium/subepicardium thickness.** A. Mice exposed to chronic hypoxia shows a bigger heart’s size in contrast to normoxic mice. Scale bar: 1mm. B. Higher ratio of heart weight to body weight in hypoxic female and male (n(female)=11 (N), 20(H), n(male)= 12 (N) and 9(H)). C. Epicardial thickness during development at E10.5, E13.5 and E17.5. EPDC migration occurs around E.13.5 (white arrowhead). 1. Epicardium, 2. Subepicardium. Scale bar: 20μm (3 top panels), 10 μm (bottom panel). D. Epicardium is highly active at E13.5. A subset of epicardial cells (EPDCs) migrate to the subepicardium. Epicardium is less active at the end of fetal gestation, E17.5. All sections were stained with DAPI to visualize the nuclei. Values are normalized by the total number of nuclei per layer and per fields and all values are represented in percentage. The line in the box plot represents the median. Statistical significance was determined by Mann-Whitney test. *****p*<0.0001, ****p*<0.001. Epic: Epicardium.

**Figure S II. Gating strategy used to distinguish the main cardiac cell types.** A. Exclusion of debris and doublets (SSC and FSC), dead cells (7AAD^-^) and hematopoietic compartment (CD45^-^Ter119^-^CD11b^-^). Epicardial cells were defined by exclusion of hematopoietic cells (CD45^+^Ter119^+^CD11b^+^), endothelial cells (CD31^+^) and stromal cells (PDGFRα^+^) and express Gp38^+^ (CD45^-^Ter119^-^CD11b^-^CD31^-^PDGFRα^-^Gp38^+^). Endothelial cells are CD31^+^ (CD45^-^Ter119^-^CD11b^-^PDGFRα^-^CD31^+^) and stromal cells are PDGFRα^+^ (CD45^-^Ter119^-^CD11b^-^CD31^-^PDGFRα^+^). B. Demonstration of the different cardiac populations that express Gp38 as a surface protein from P0 and Ad. P0: postnatal day 0, Ad: Adult, BVE: Blood vessel endothelial cell, LVE: lymphatic vessel endothelial cell, StrC: stromal cell, Epic: epicardium.

**Figure S III. The overall expression of Pw1 decrease throughout life.** A. Coronal heart sections show the decrease of Xgal-PW1 expression from P0 to adult mice and its upregulation upon *in vivo* hypoxia exposure. Scale bar: 1mm. B. Flow cytometry analysis correlates the decrease in PW1-FDG expression along time (P0 to adult mice) and confirms the upregulation upon *in vivo* hypoxia exposure. C. Myocardium show lower frequency of PW1^+^ cells as compared to the epicardium/subepicardium (n=3, 20 images per heart and per region). D. Increase of β-gal (PW1) and Wt1 co-expression in the subepicardium and myocardium. (n=3, 20 images per heart and per region). E. Absolute numbers of proliferative cells in hypoxic mice (n=3, 20 images per heart and per region) in Epic. F. Absolute numbers of the increase of WT1 expression in the epicardial, subepicardial and endothelial cells in hypoxic mice (n=3, 20 images per heart and per region).

**Figure S IV. Transcriptional profiling at the population level.** A. Multiplex qPCR heat-map at the population level to compare the transcriptional profile of epicardial cells (Gp38^+^), epicardial-derived/stromal cells (PDGFRα^+^) and endothelial cells (CD31^+^) from E17.5, P0, Ad and H-Ad. Each column represents an independent-sorted cell subset (100 cells/population) with the corresponding color-code according to cell type and physiological state (minimum n=4 per population). Gene expression was normalized to *Gapdh*, and unsupervised hierarchical clustering was performed. B. tSNE analysis and hierarchical clustering of multiplex qPCR data at the population level (minimum n=4 per population). C. Relative expression of *Upk1b* and *Upk3b* genes in epicardial cells (Gp38^+^), epicardial-derived/stromal cells (PDGFRα^+^) and endothelial cells (CD31^+^) from P0 hearts. Color-code according to the cell type (top panel) and according to the physiological state (bottom panel). E17.5: embryonic day 17.5, P0: postnatal day 0, Ad: Adult, H: Hypoxia, EC: endothelial cell, Dev: developing, Epic: epicardium.

**Figure S V. Transcriptional profiling at the single cell level.** A. Multiplex qPCR heat-map at single cell level to compare the transcriptional profile of epicardial cells (Gp38^+^), stromal cells (PDGFRα^+^) and endothelial cells (CD31^+^) from E17.5, P0, Ad and H-Ad. Each column represents one cell with the corresponding color-code according cell type and physiological state. A total of 382 cells were analyzed. Gene expression was normalized to *Gapdh*, and unsupervised hierarchical clustering was performed. B. tSNE analysis and hierarchical clustering of multiplex qPCR data at single cell level (382 cells). C. Diffusion map (pseudo-time analysis) of Sc, Ec, and Smc populations based on single cell gene expression. Color-code according to the cell type (left panel) and according to the physiological state (right panel). E17.5: embryonic day 17.5, P0: postnatal day 0, Ad: Adult, H: Hypoxia. EC: endothelial cell, Epic: epicardium.

**Figure S VI. Epicardial and endothelial gene expression quantification in the Gp38^+^ epicardial cells.** A. Relative epicardial gene expression in Gp38^+^ cells from P0, Ad and H-Ad. B. Relative endothelial gene expression in Gp38^+^ cells from P0, Ad and H-Ad. Color-code according to the physiological state (right panel). P0: postnatal day 0, Ad: Adult, H: Hypoxia.

**Figure S VII. Early-stage characterization of the developing colonies.** A. Immunolabeling of the cell cycle makers Ki67 and pH3 compares the proliferative profile of colony both on D4 and D8 after culture (self-renewal). B. Immunolabeling of the Gp38^+^ colony showing the colony keeps the expression of Gp38 and PW1 (clonogenicity) at D4 after culture. C. Immunolabeling of the Gp38^+^ colony showing smooth muscle cell markers (αSMA and SM22α) are observed at early stage. D. Endothelial marker Flk1 is observed at early stage of the Gp38^+^ colony, but not CD31. Scale bar: 100μm. All nuclei were counterstained with DAPI**.**

**Figure S VIII. Detailed characterization of the developing colonies *in vitro*.**

Immunolabeling detailed in split channels for: SM22α, Gp38, αSMA, Ki67, pH3, PW1, PDGFRα, Flk1, CD31 and DAPI.
